# *Ent*-Abietane Diterpenoids from *Euphorbia fischeriana* and Their Cytotoxic Activities

**DOI:** 10.3390/molecules27217258

**Published:** 2022-10-26

**Authors:** Qin-Feng Zhu, Guo-Bo Xu, Shang-Gao Liao, Xue-Long Yan

**Affiliations:** School of Pharmacy, Guizhou Medical University, Dongqing Road, Guiyang 550025, China

**Keywords:** abietane-type, diterpenoid, *Euphorbia fischeriana*, antiproliferative activities

## Abstract

The roots of *Euphorbia fischeriana* have been used as a traditional Chinese medicine for the treatment of tuberculosis and ringworm. In the current study, diterpenoids from the ethyl acetate extract of the roots *E. fischeriana* and their cytotoxic effects against five cancer lines were investigated. Two new *ent*-abietane diterpenoids, euphonoids H and I (**1**–**2**), as well as their two analogues (**3**–**4**) were first isolated from this source. The structures of the two new compounds were elucidated on the basis of spectroscopic data and quantum chemical calculation. Their absolute configurations were assigned via ECD spectrum calculation. The isolated compounds were evaluated for their antiproliferative activities against five cancer cell lines. Compounds **1** and **2** exhibited significant inhibitory effects against human prostate cancers C4-2B and C4-2B/ENZR cell lines with IC_50_ values ranging from 4.16 ± 0.42 to 5.74 ± 0.45 μM.

## 1. Introduction

Natural products are promising sources for the discovery of novel agents/active templates for the development of effective agents against a variety of human diseases [1]. Due to their great structural diversity and wide range of bioactivities [2], diterpenoids have been a constant focus of drug discovery. Among the naturally occurring cyclic diterpenoids (e.g., abietane, labdane and clerodane diterpenoids), tricyclic abietane diterpenoids are of particular significance. These diterpenoids were reported to be present in species of Lamiaceae, Cupressaceae, Pinaceae and Euphorbiaceae as well as in several higher plants [3,4] and were shown to possess antitumor, antioxidant, antibacterial and anti-inflammatory effects [5]. Sugiol, an abietane diterpenoid previously isolated from *Metasequoia glyptostroboides* (Cupressaceae), has been developed as an advanced multimodal anti-inflammatory disease targeting tool [6]; Euphelionolides A, D, I and L, four *ent*-abietane diterpenoids isolated from *Euphorbia helioscopia* (Euphorbiaceae), were demonstrated to be effective free-radical scavengers acting via various reaction pathways [7]; 6-hydroxy-5,6-dehydrosugiol, a derivative of sugiol isolated from the stem bark of *Cryptomeria japonica*, was shown to be a potent androgen receptor antagonist in PCa cells [8].

The roots of *Euphorbia fischeriana* Steud have been used as a traditional Chinese medicine for the treatment of lymphoid tuberculosis and ringworm [9]. Previous phytochemical studies showed that polycyclic diterpenoids including *ent*-abietanes, *ent*-atisanes, *ent*-kauranes, *ent*-isopimaranes and *ent*-pimaranes possessing a common 6/6/6-tricyclic ring are the major constituents of *E. fischeriana* [10,11]. In our earlier study, a series of *ent*-abietane diterpenoids with significant cytotoxicity have been isolated from this plant [12]. As part of our continuing efforts toward novel antitumor diterpenoids, the chemical constituents of the roots of *E. fischeriana* were reinvestigated. As a result, two previously undescribed *ent*-abietanes and two known analogues were isolated from the roots of *E. fischeriana*. The new compounds showed significant cytotoxicity against human prostate cancer C4-2B and C4-2B/ENZR cell lines. Herein, the details of isolation, structure elucidation and cytotoxicity of these compounds are described.

## 2. Results and Discussion

**Figure 1:**
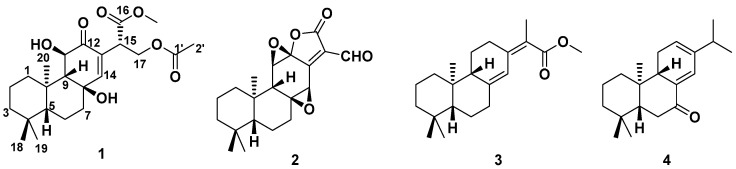
The structures of compounds **1**–**4**.

The dry roots of *E. fischeriana* were repeatedly extracted with 95% EtOH at room temperature and the extract was successively partitioned with petroleum ether, ethyl acetate (EtOAc) and *n*-butanol. The EtOAc fraction was subjected repeatedly to column chromatography over silica gel, ODS gel, Sephadex LH-20 and semipreparative HPLC to yield two previously undescribed *ent*-abietane diterpenoids (**1**–**2**). In addition to the above new compounds, two known diterpenoids (Figure 1) were also obtained: raserranes A (**3**) and B (**4**). The structures of all compounds were well-characterized by NMR analysis and quantum chemical calculation.

### 2.1. Structure Elucidation of Compounds ***1*** and ***2***

Euphonoid H (**1**) was obtained as a colorless oil and was shown to possess a molecular formula of C_23_H_34_O_7_ based on its HRESIMS ion at 445.2213 [M + Na]^+^ (calcd 445.2197). The ^1^H spectrum (Supplementary Material) showed signals for an olefinic hydrogen (δ_H_ 6.71, H-14), one methoxyl (δ_H_ 3.68, 16-OCH_3_) and four methyl groups (δ_H_ 2.02, 0.91, 0.79 and 0.69). The ^13^C and HSQC NMR showed resonances assignable to one ketone (δ_C_ 196.0), two ester (δ_C_ 170.8 and 171.1), two olefinic carbons (δ_C_ 133.7 and 154.2), four methyl (δ_C_ 34.0, 22.1, 17.8 and 21.0), one methoxyl group (δ_C_ 34.0), three oxygenated carbons (δ_C_ 72.0, 69.5 and 62.5) and ten additional sp^3^ carbons (δ_C_ between 18.5 and 60.4). Comprehensive analysis of the 1D and 2D-NMR data (Table 1) revealed that compound **1** possessed, except for an acetoxyl group and a methoxy group, an abietane diterpene skeleton similar to that of methyl-8β,11β-dihydroxy-12-oxo-ent-abietadi-13,15(17)-ene-16-oate previously isolated from this plant [13]. However, the ^13^C NMR data for the Δ^15(17)^ (δ_C_ 137.3 for C-15 and 128.8 for C-17) of the latter were replaced by signals for a methine (δ_C_ 45.1, C-15) and an oxymethylene (δ_C_ 62.5, C-17). These observations implied that compound **1** was a hydrogenated derivative of the known compound.

**Figure 2:**
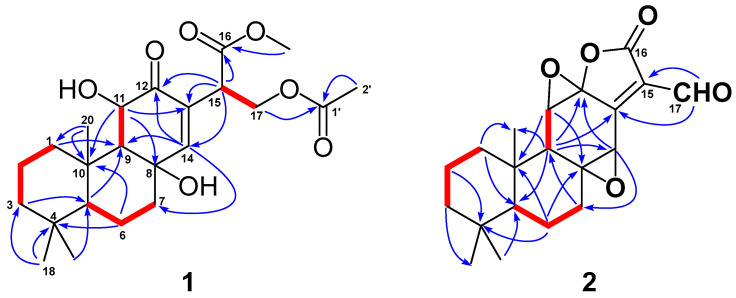
Key ^1^H-^1^H COSY (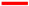) and HMBC (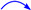) correlations of **1** and **2**.

Detailed 2D-NMR (^1^H-^1^H COSY, HSQC, HMBC and NOESY) data analysis further confirmed the above deduction and fulfilled the structural assignment. The ^1^H-^1^H COSY revealed four spin systems, CH_2_-1/CH_2_-2/CH_2_-3, H-5/CH_2_-6/CH_2_-7, H-9/H-11 and H-15/CH_2_-17 (Figure 2). HMBC correlations from H_3_-20 (to C-1 and C-10), H_2_-1 (to C-9), H_3_-18 (to C-3, C-4 and C-5), H_2_-6 (to C-4 and C-10), H-11 (to C-8 and C-13) and H-14 (to C-7, C-9 and C-12) to their corresponding carbons not only connected the former three fragments, but suggested that compound **2** shared the same ABC rings with methyl-8β,11β-dihydroxy-12-oxo-ent-abietadi-13,15(17)-ene-16-oate. In addition, HMBC correlations from H-15 to C-12, C-13 and C-14 and from H_2_-17 to C-13 located the Δ^15(17)^ double bond at C-13. HMBC correlations from the methoxyl group to C-16 suggested the presence of a methoxyformyl group, while the HMBC correlation from H_2_-17 to C-16 revealed its position at C-15. The acetoxyl group was connected to the abietane skeleton at C-17 by the key HMBC cross-peaks from H_2_-17 and H_3_-2′ to C-1′. Thus, the gross structure of **1** was established as depicted.

**Figure 3:**
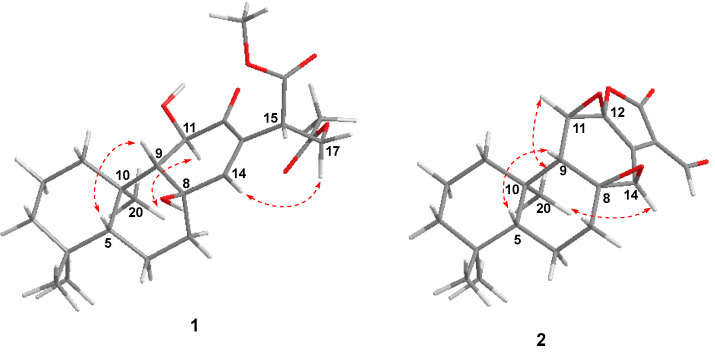
Key NOE correlations (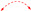) of compounds **1** and **2**.

**Figure 4:**
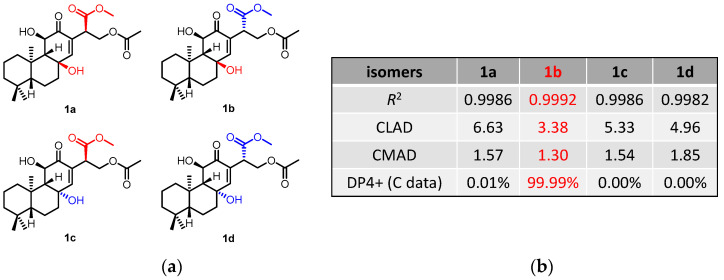
^13^C NMR calculation results of compound **1** at the mPW1PW91/6-311+G(d,p) level. (**a**) Structures of conformer **1a**–**1d**. (**b**) Key parameters of the calculated chemical shifts of conformers **1a**–**1d**.

The NOESY correlations (Figure 3) of H-5/H-9 indicated that these protons were cofacial and were arbitrarily assigned to be *β*-oriented, while the NOESY correlation of H_3_-20/H-11 indicated that these protons were *α*-oriented. However, the NOESY spectrum did not give useful signals to determine the relative configuration of C-8 and C-15. To establish the relative configuration, the chemical shifts of four conformers were predicted at the B3LYP/6-311+G (d, p) level in chloroform (Figure 4). The results showed that the calculated chemical shifts of conformer **1b** was in the best agreement with the experimental values among those predicted for **1a**, **1b**, **1c** and **1d**. Further DP4+ analyses verified that conformer **1b** was assigned with a 99.99% probability among all the conformers (Figure 4). These results suggested that compound **1** had the structure of conformer **1b** with the relative stereochemistry of 5R*, 8R*, 9R*, 10R*, 11R*, 15S*.

**Figure 5:**
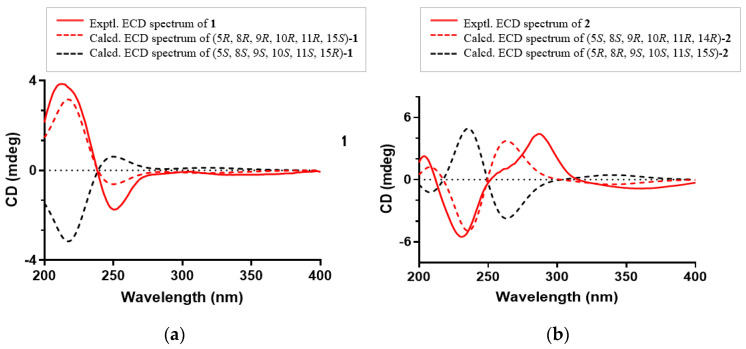
(**a**) Experimental and calculated ECD spectra of **1**; (**b**) Experimental and calculated ECD spectra of **2**.

The absolute configuration of **1** was established by comparing its experimental ECD spectrum with those calculated at the CAM-B3LYP/6-31+G(d) level in acetonitrile. As shown in Figure 5, the experimental ECD curve of **1** showed first negative and second positive Cotton effects around 250 and 213 nm, respectively, which matched well the calculated ECD spectrum of 5*R*, 8*R*, 9*R*, 10*R*, 11*R*, 15*S-***1** (Figure 5a). Thus, compound **1,** as an *ent*-abietane diterpenoid, was established as depicted and named euphonoid H.

**Figure 6:**
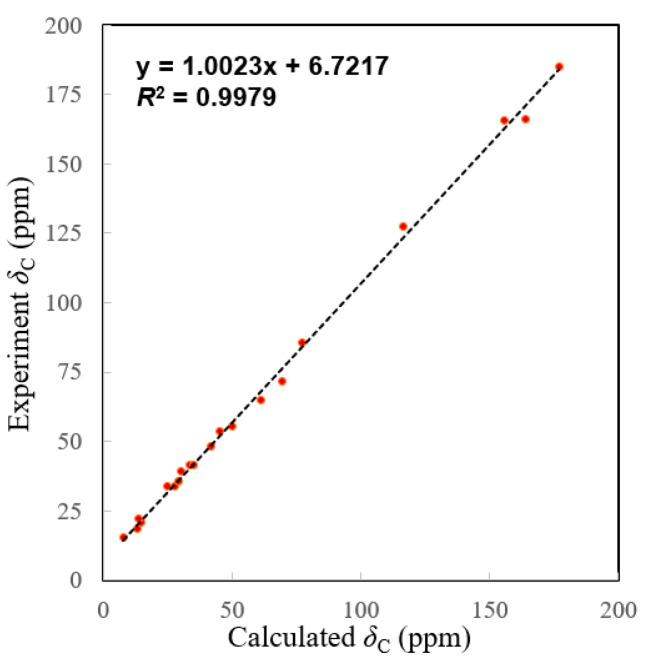
Linear correlation plots of predicted versus experimental ^13^C NMR chemical shifts.

The molecular formula of **2** was determined to be C_20_H_24_O_5_ by the HRESIMS ion peak at *m*/*z* 343.1550 ([M − H]^−^, calcd 343.1551). The ^1^H NMR data (Table 1) of **2** indicated the presence of three methyls [*δ*_H_ 0.98 (3H, Me-18), 0.85 (3H, Me-19), 0.80 (3H, Me-20)] and one aldehyde [*δ*_H_ 9.97 (s, H-17)]. The ^13^C NMR (Table 1) and HSQC data of **2** revealed the presence of three methyls, five methylenes, five methines (including two oxygenated ones at *δ*_C_ 64.8 and 55.3 and seven quaternary carbons (including two olefinic ones at *δ*_C_ 166.2 and 127.5). The above NMR characteristic features of **2** resembled those of jolkinolide B [14], the major differences being the replacement of the 17-CH_3_ group in jolkinolide B by an aldehyde group (*δ*_C_ 185.0) in **2**. HMBC correlations from H-17 (*δ*_H_ 9.97, s) to C-13 (*δ*_C_ 166.2) and C-15 (*δ*_C_ 127.5) further confirmed the above deduction. The NOESY correlation H-5/H-9 suggested that H-5 and H-9 were *β*-oriented, whereas the NOESY correlations H_3_-20/H-11 and H_3_-20/H-14 indicated that H-11, H-14 and CH_3_-20 were *α*-oriented. Subsequently, quantum chemical calculation of NMR chemical shifts was run on the proposed structure of **2**. As indicated by R^2^ (^13^C: 0.9979), CMAD (^13^C: 1.88 ppm) and CLAD (^13^C: 4.79 ppm) good consistency was observed between the theoretically predicted and experimental chemical shifts, which validated the proposed structure for **2** (Figure 6). Subsequently, ECD calculation (Figure 5b) of the two enantiomers of **2** enabled the establishment of the absolute configuration of **2** to be 5*S*, 8*S*, 9*R*, 10*R*, 11*R*, 12*R*, 14*R*. The structure of **2** was therefore established as depicted and named euphonoid I.

The other two known diterpenoids (**3**–**4**) were identified to be *ent*-abietane diterpenoids raserranes A (**3**) and B (**4**) by comparison of their NMR data with those reported in the literature, these four diterpenoids were discovered for the first time from this species [15].

### 2.2. Biological Activity of Isolated Compounds

The anticancer effects of the isolates **1**–**4** were evaluated against human breast cancer cells MDA-MB-231, human colon cancer cells HCT-15 and RKO and human prostate cancer cells C4-2B and C4-2B/ENZR (enzalutamide-resistant C4-2B cells). The IC_50_ values (Table 2) indicated that the two new compounds exhibited varying degrees of growth inhibition against the five cancer cell lines. Compound **1** showed significant inhibitory activities against C4-2B and C4-2B/ENZR cell lines with IC_50_ values of 5.52 ± 0.65 µM and 4.16 ± 0.42 µM, respectively. Compound **2** exhibited marked inhibitory activity towards the five human cancer cell lines (IC_50_ values ranging from 4.49 ± 0.78 to 12.45 ± 3.24 µM) and was particularly active against C4-2B and C4-2B/ENZR cell lines (IC_50_ values: 4.49 ± 0.78 and 5.74 ± 0.45, respectively).

Macrocyclic and polycyclic diterpenes were usually encountered in the genus of *Euphorbia* and macrocyclic diterpenes were characteristic components of *Euphorbia* plants, while polycyclic diterpenes were nonspecific in this genus. Although polycyclic diterpenes were not the characteristic components of *Euphorbia* plants, some polycyclic diterpenes showed great potential in the development of anticancer drugs [16,17,18]. Jolkinolide B, a typical *ent*-abietane diterpene first isolated from *Euphorbia jolkini*, induced apoptosis and sensitized bladder cancer to mTOR inhibitors [19,20]; 17-hydroxy-jolkinolide B, a potent inhibitor of JAK/STAT3 signaling, is a promising anticancer drug candidate [21]. In this study, compounds **1**–**2** sharing the same abietane diterpene skeleton (6/6/6 carbon ring system) were shown to be promising anti-prostate cancer candidates. Among the four compounds isolated, compound **2** that possessed an α,β-unsaturated γ-lactone ring at C-12 and C-13, was very active against almost the test cancer cells. This observation was consistent with our previous discovery that such an α,β-unsaturated γ-lactone ring was beneficial for the anticancer activity of this type of diterpenoids [12]. Despite the fact that several antitumor abietane diterpenoids were reported in recent years, the pharmacophores and structure-activity relationship of abietane diterpenoids as anticancer agents were rarely investigated. Thus, synthesis of these diterpenoids and study of their structure–activity relationship and potential molecular mechanisms were of great significance for the design and development of anticancer agents.

## 3. Materials and Methods

### 3.1. General Experimental Procedures

Optical rotations were carried out on a Rudolph Autopol I automatic polarimeter (Rudolph Research Analytical, Hackettstown, NJ, USA). The UV spectra were measured at a Shimadzu UV-2450 spectrophotometer (Shimadzu Corporation, Kyoto, Japan). IR spectra were determined on a Bruker Tensor 37 infrared spectrophotometer (Bruker Optics, Ettlingen, Germany) with KBr disk. ECD spectra were measured on an Applied Photophysics Chirascan spectrometer (Applied Photophysics Ltd., England). NMR spectra were measured on Bruker AM-400 spectrometer with tetramethylsilane (TMS) as the internal standard. HR-ESIMS data were determined using a Waters Micromass Q-TOF spectrometer (Waters Corporation, Milford, MA, USA). The semi-preparative HPLC was performed on an Essentia LC-16 (Shimadzu, Suzhou, China). Column chromatography (CC) was used using silica gel (200–300 mesh, Qingdao Marine Chemical Factory, Qingdao, China).

### 3.2. Plant Material

The roots of *E. fischeriana* were collected in August 2015 from Tie ling city, Liaoning Province, P. R. China and identified by Prof. Qing-De Long (Guizhou Medical University). The specimens were deposited in School of Pharmaceutical Sciences, Guizhou Medical University (specimen no. 20150805).

### 3.3. Extraction and Isolation

The dried roots (10 kg) of *E. fischeriana* were crushed, extracted with 95% ethanol (50 L) at room temperature for three times (each for 24 h), the solvent was recovered under reduced pressure to obtain the crude extract. The crude extract was suspended in water (3 L) and then partitioned sequentially with petroleum ether, EtOAc and *n*-BuOH (saturated with water) and dried under reduced pressure to give their corresponding extracts (178.36, 220.82 and 205.43 g, respectively). The EtOAc fraction was subjected to CC (chromatographic column) on silica gel (1.3 kg, 100–200 mesh) using petroleum ether-CH_2_Cl_2_ (1:0 to 0:1) and CH_2_Cl_2_-MeOH (200:1 to 10:1) as eluents to give fractions 1–3 and 4–10, respectively. Fr. 5 (12.15 g) showed obvious brick-red spots in the TLC and was subjected to ODS CC eluted with MeOH-H_2_O (40–100%) to obtain 6 fractions (Fr. 5A to Fr. 5F). Fr. 5C (2.42 g) was subsequently loaded on a Sephadex LH-20 column using CH_2_Cl_2_-MeOH (1:1) as eluent to obtain 3 fractions (Fr. 5C-a to Fr. 5C-c). Fr. 5C-a (0.69 g) was purified by semipreparative HPLC (ACN-H_2_O, 87%, 3 mL/min) to obtain compound **3** (6.8 mg, t_R_ = 12.0 min). Compound **1** (4.3 mg, t_R_ = 18.5 min) was isolated from Fr. 5C-b (0.75 g) by preparative HPLC (ACN-H_2_O, 85%, 3 mL/min). Fr. 5D (1.87 g) was firstly separated by silica gel CC (PE/EtOAc, 5:1) and then by semi-HPLC (ACN-H_2_O, 90%, 3 mL/min) to afford **2** (3.5 mg, t_R_ = 13.2 min) and **4** (7.2 mg, t_R_ = 16.7 min), respectively.

Euphonoid H (**1**): colorless oil; ${\left[\alpha \right]}_{D}^{20}$ −43.2 (c 0.10, CHCl_3_); UV (MeOH) *λ*_max_ (log ε) 230 (3.68) nm; IR (KBr) *ν*_max_ 3400, 2932, 1738, 1217, 1033 cm^−1^; HRESIMS *m*/*z* 445.2213 (calcd. for C_23_H_34_O_7_Na^+^ [M + Na]^+^, 445.2197); ^1^H and ^13^C NMR data see Table 1.

Euphonoid I (**2**): colorless oil; ${\left[\alpha \right]}_{D}^{20}$ + 2.8 (c 0.37, CHCl_3_); UV (MeOH) λ_max_ (log ε) 266 (4.39) nm; IR (KBr) *ν*_max_ 2938, 1788, 1636, 1256, 968 cm^−1^; HRESIMS *m*/*z* 343.1550 (calcd. for C_20_H_23_O_5_^-^ [M − H]^−^, 343.1551); ^1^H and ^13^C NMR data see Table 1.

Raserrane A (**3**): colorless oil; ${\left[\alpha \right]}_{D}^{20}$ −42.8 (c 0.10, CHCl_3_); HRESIMS *m*/*z* 339.2291 (calcd. for C_21_H_32_O_2_Na^+^ [M + Na]^+^, 339.2295).

Raserrane B (**4**): colorless oil; ${\left[\alpha \right]}_{D}^{20}$ −133.4 (c 0.25, CHCl_3_); HRESIMS *m*/*z* 287.2362 (calcd. for C_20_H_31_O^+^ [M + H]^+^, 287.2369).

### 3.4. Quantum Chemical NMR and ECD Calculations of Compound ***1***–***2***

The random conformational searches were performed by SYBYL X 2.1.1 program using MMFF94s molecular force field. The obtained conformers were subsequently optimized by using Gaussion09 software at the B3LYP/6-31G(d) level in gas phase. The optimized stable conformers were selected for further NMR calculations at the mPW1PW91/6-311 + G(d,p) level in chloroform and ECD calculations at the CAM-B3LYP/6-31 + G(d) level in acetonitrile. The overall theoretical NMR data were analyzed by using linear regression and DP4+ probability. The overall ECD data were weighted by Boltzmann distribution and produced by SpecDis version 1.70.1 software (T. Bruhn; A. Schaumlöffel; Y. Hemberger; G. Pescitelli, Berlin, Germany).

### 3.5. Cell Culture

Five cancer cell lines, including human prostate cancer cells (C4-2B), enzalutamide-resistant C4-2B cells (C4-2B/ENZR), human breast cancer cells (MDA-MB-231) and human colon cancer cells (HCT-15 and RKO) used in this study were purchased from the Laboratory Animal Service Centre at Sun Yat-sen University (Guangzhou, China). Cell lines were cultured in Dulbecco’s modified Eagle’s medium (DMEM) with 10% fetal bovine serum (FBS) and antibiotics (100 units/mL penicillin and 100 g/mL streptomycin). These cells were incubated at 37 °C in an atmosphere of 5% CO_2_.

### 3.6. Cytotoxicity Assay

The cells in logarithmic growth phase were seeded a in 96-well plates at a density of 5 × 10^3^ cells/well for 24 h. Then, cells were treated with different concentrations of the compounds for an additional 48 h. Subsequently, 10 μL MTT (5 mg/mL) (Sigma, Saint Louis, MO, USA) were added to each well. After incubation in the incubator for 4 h, the suspension was discarded and the dark blue crystals were solubilized in dimethyl sulfoxide (DMSO). The absorbance of the solution was detected by a multifunction micro-plate reader (Molecular Devices, Flex Station 3, Molecular Devices, San Francisco, USA) at 450 nm. IC_50_ value was used to express the cytotoxic effect on the tested compounds.

## 4. Conclusions

In summary, two new highly oxygenated *ent*-abietane diterpenoids euphonoids H and I (**1**–**2**), together with two known analogues raserranes A (**3**) and B (**4**) were separated and identified from the EtOAc-soluble partition of the roots of *E. fischeriana*. Their structures were elucidated by comprehensive spectroscopic analysis, quantum chemical calculation and ECD calculations. All the compounds were isolated from *E. fischerian* for the first time. The two new compounds exhibited strong antiproliferative potency against the human prostate cancer cells C4-2B and C4-2B/ENZR, with IC_50_ values less than 10 μM. This study not only enriches the chemical diversity of *ent*-abietane diterpenoids in the *Euphorbia* species but also forms a basis for the discovery of bioactive natural products from Euphorbiaceae herbs.

The current results, together with others’ previous discoveries, suggested that *ent*-abietane diterpenoids with certain structural motifs might possess very strong anticancer activity against prostate cancer cell lines and this type of diterpenoids provided a promising skeleton for the development of anti-cancer agents for the treatment of prostate cancers.

## Figures and Tables

**Table 1:** ^1^H (400 MHz) and ^13^C (100 MHz) NMR data for **1** and **2** in CDCl_3_.

Position	1		2	
	*δ*_H_ (*J* in Hz)	*δ*_C_, Type	*δ*_H_ (*J* in Hz)	Δ_c_, Type
1	1.88 (1H, d, *J* = 13.0 Hz) 1.21–1.16 (1H, m)	39.7, CH_2_	1.92 (1H, d, *J* = 12.5 Hz) 1.37–1.29 (1H, m)	39.2, CH_2_
2	1.55 (1H, d, *J* = 13.5 Hz) 1.51–1.45 (1H, m)	18.5, CH_2_	1.63–1.55 (1H, m) 1.56–1.51 (1H, m)	18.5, CH_2_
3	1.43 (1H, d, *J* = 12.7 Hz) 1.26–1.19 (1H, m)	41.6, CH_2_	1.47–1.43 (1H, m) 1.29–1.20 (1H, m)	41.4, CH_2_
4		33.3, C		33.6, C
5	1.08 (1H, dd, *J* = 12.9, 2.6 Hz)	54.4, CH	1.12 (1H, dd, *J* = 12.3, 2.5 Hz)	53.6, CH
6	1.71–1.65 (1H, m) 1.16–1.12 (1H, m)	19.2, CH_2_	1.88–1.80 (1H, m) 1.56–1.51 (1H, m), overlapped	20.9, CH_2_
7	2.13–2.03(1H, m) 1.74–1.72 (1H, m)	41.4, CH_2_	2.08–1.97 (1H, m) 1.51–1.48 (1H, m), overlapped	35.8, CH_2_
8		69.5, C		71.7, C
9	2.13–2.03(1H, s)	60.4, CH	2.33 (1H, s)	48.1, CH
10		37.7, C		39.4, C
11	4.23 (1H, s)	72.0, CH	4.15 (1H, s)	64.8, CH
12		196.0, C		85.4, C
13		133.7, C		166.2, C
14	6.71 (1H, s)	154.2, CH	4.48 (1H, s)	55.3, CH
15	3.77 (1H, t, *J* = 7.2 Hz)	45.7, CH		127.5, C
16		170.8, C		165.8, C
17	4.41 (2H, d, *J* = 7.2 Hz)	62.5, CH_2_	9.97 (1H, s)	185.0, CH
18	0.91 (3H, s)	34.0, CH_3_	0.94 (3H, s)	33.6, CH_3_
19	0.79 (3H, s)	22.1, CH_3_	0.85 (3H, s)	22.0, CH_3_
20	0.69 (3H, s)	17.8, CH_3_	0.80 (3H, s)	15.6, CH_3_
16-OCH_3_	3.68 (3H, s)	52.4, CH_3_		
1′		171.1, C		
2′	2.02 (3H, s)	21.0, CH_3_		

**Table 2:** IC_50_ data of compounds **1**–**4** for the indicated cell lines.

Compound	IC_50_^1^ (µM)				
	MDA-MB-231	HCT-15	RKO	C4-2B	C4-2B/ENZR
**1**	21.80 ± 2.35	28.57 ± 1.16	20.46 ± 1.43	5.52 ± 0.65	4.16 ± 0.42
**2**	7.95 ± 0.82	12.45 ± 3.24	8.78 ± 2.45	4.49 ± 0.78	5.74 ± 0.45
**3**	>50	>50	>50	34.09 ± 7.78	>50
**4**	>50	>50	>50	23.34 ± 2.18	36.98 ± 6.18
DOX ^2^	0.34 ± 0.16	0.72 ± 0.09	0.59 ± 0.29	0.11 ± 0.08	0.22 ± 0.18

^1^ Each IC_50_ value was the mean ± standard deviation from three experiments; ^2^ Positive control: doxorubicin.

## Data Availability

The data are available in the Supporting Information of the article.

## Supplementary Materials

The following supporting information can be downloaded online: https://www.mdpi.com/article/10.3390/molecules27217258/s1, Figures S1–S6: The 1D and 2D NMR (400 MHz) spectra of compound **1** in CDCl_3_, Figure S7: The HRESIMS spectrum of compound **1**, Figure S8: The IR spectrum of compound **1**, Figures S9–S14: The 1D and 2D NMR (400 MHz) spectra of compound **2** in CDCl_3_, Figure S15: The HRESIMS spectrum of compound **2**, Figure S16: The IR spectrum of compound **2**, Figures S17 and S18 and Tables S1–S9: NMR and ECD calculation method of compound **1**, Figures S19–S21 and Tables S10–S12: NMR and ECD calculation method of compound **2**, Figures S22–S25: ^1^H NMR and HRESIMS spectra of compound **1**–**2**, Table S13: NMR Data for compounds **1**–**2** in CDCl_3_.

## Sample Availability

Samples of the compounds are not available from the authors as the material has been used up for bioassays.

## References

[1] Newman D.J., Cragg G.M. (2020). Natural products as sources of new drugs over the nearly four decades from 01/1981 to 09/2019. J. Nat. Prod..

[2] Hanson J.R. (2009). Diterpenoids. Nat. Prod. Rep..

[3] Jian B., Zhang H., Liu J. (2018). Structural diversity and biological activities of diterpenoids derived from *Euphorbia fischeriana* steud. Molecules.

[4] Wu Y.-B., Ni Z.-Y., Shi Q.-W., Dong M., Kiyota H., Gu Y.-C., Cong B. (2012). Constituents from *Salvia* species and their biological activities. Chem. Rev..

[5] Zhao H., Sun L., Kong C., Mei W., Dai H., Xu F., Huang S. (2022). Phytochemical and pharmacological review of diterpenoids from the genus *Euphorbia* Linn (2012–2021). J. Ethnopharmacol..

[6] Bajpai V.K., Sonwal S., Hwang S.-K., Shukla S., Khan I., Dey D.K., Chen L., Simal-Gandara J., Xiao J., Huh Y.S. (2021). Sugiol, a diterpenoid: Therapeutic actions and molecular pathways involved. Pharmacol. Res..

[7] Ngo T.C., Dao D.Q., Mai T.V.T., Nguyen T.L.A., Huynh L.K. (2022). On the radical scavenging and DNA repairing activities by natural oxygenated diterpenoids: Theoretical insights. J. Chem. Inf. Model..

[8] Lin F.-M., Tsai C.-H., Yang Y.-C., Tu W.-C., Chen L.-R., Liang Y.-S., Wang S.-Y., Shyur L.-F., Chien S.-C., Cha T.-L. (2008). A novel diterpene suppresses CWR22Rv1 tumor growth in vivo through antiproliferation and proapoptosis. Cancer Res..

[9] Chinese Pharmacopoeia Commission (2020). Pharmacopoeia of the People’s Republic of China.

[10] Shi Q.-W., Su X.-H., Kiyota H. (2008). Chemical and pharmacological research of the plants in genus *Euphorbia*. Chem. Rev..

[11] Vasas A., Hohmann J. (2014). Euphorbia diterpenes: Isolation, structure, biological activity, and synthesis (2008–2012). Chem. Rev..

[12] Yan X.-L., Zhang J.-S., Huang J.-L., Zhang Y., Chen J.-Q., Tang G.-H., Yin S. (2019). Euphonoids A−G, cytotoxic diterpenoids from *Euphorbia fischeriana*. Phytochemistry.

[13] Morgenstern T., Bittner M., Silva M., Aqueveque P., Jakupovic J. (1996). Diterpenes and phloracetophenones from *Euphorbia portulacoides*. Phytochemistry.

[14] Uemura D., Hirata Y. (1972). Two new diterpenoids, jolkinolides A and B, obtained from *Euphorbia jolkini* boiss. (Euphorbiaceae). Tetrahedron Lett..

[15] Liu G.-L., Xu W., Liu X.-J., Yan X.-L., Chen J. (2018). Two new abietane diterpenoids from the leaves of *Rabdosia serra*. J. Asian Nat. Prod. Res..

[16] Jian B.-Y., Zhang H., Han C.-C., Liu J.-C. (2018). Anti-cancer activities of diterpenoids derived from *Euphorbia fischeriana* Steud. Molecules.

[17] Rosaria A., Giuseppe A.M., Monica R.L., Xiao J.-B., Simone B., Rosa T. (2022). Advances on natural abietane, labdane and clerodane diterpenes as anti-cancer agents: Sources and mechanisms of action. Molecules.

[18] Yan X.-L., Zou M.-F., Chen B.-L., Yuan F.-Y., Zhu Q.-F., Zhang X., Lin Y., Long Q.-D., Liu W.-L., Liao S.-G. (2022). Euphorane C, an unusual C17-norabietane diterpenoid from *Euphorbia dracunculoides* induces cell cycle arrest and apoptosis in human leukemia K562 cells. Arab. J. Chem..

[19] Sang J., Li W., Diao H.-J., Fan R.-Z., Huang J.-L., Gan L., Zou M.-F., Tang G.-H., Yin S. (2021). Jolkinolide B targets thioredoxin and glutathione systems to induce ROS-mediated paraptosis and apoptosis in bladder cancer cells. Cancer Lett..

[20] Sang J., Gan L., Zou M.-F., Lin Z.-J., Fan R.-Z., Huang J.-L., Li W., Tang G.-H., Yin S. (2022). Jolkinolide B sensitizes bladder cancer to mTOR inhibitors via dual inhibition of Akt signaling and autophagy. Cancer Lett..

[21] Wang Y., Ma X.-Q., Yan S.-S., Shen S.-S., Zhu H.-L., Gu Y., Wang H.-B., Qin G.-W., Yu Q. (2009). 17-hydroxy-jolkinolide B inhibits signal transducers and activators of transcription 3 signaling by covalently cross-linking Janus kinases and induces apoptosis of human cancer cells. Cancer Res..

