# Supplementary material for: Ent-Abietane Diterpenoids from Euphorbia fischeriana and Their Cytotoxic Activities
Source: Molecules. 2022 Oct 26;27(21):7258. doi: 10.3390/molecules27217258 (PMC9653608; doi:10.3390/molecules27217258)
Supplement: Supplementary file 1 [file molecules-27-07258-s001.zip › molecules-1969051-supplementary.pdf]

Ent–abietane Diterpenoids from *Euphorbia fischeriana* and Their Cytotoxic Activities

Qin–Feng Zhu†, Guo–Bo Xu†, Shang–Gao Liao* and Xue–Long Yan*

School of Pharmacy, Guizhou Medical University, Dongqing Road, Guiyang, 550025, China
* Correspondence: lshangg@163.com (S.–G. L); yanxlong54@163.com (X.–L. Y).
† These authors contributed equally to this work.

Contents	Page
**Figure S1**. ^{1}H NMR spectrum of **1** in $CDCl_3$	3
**Figure S2**. ^{13}C NMR and DEPT spectrum of **1** in $CDCl_3$	3
**Figure S3**. HSQC spectrum of **1** in $CDCl_3$	4
**Figure S4**. HMBC spectrum of **1** in $CDCl_3$	4
**Figure S5**. ^{1}H−^{1}H COSY spectrum of **1** in $CDCl_3$	5
**Figure S6**. NOESY spectrum of **1** in $CDCl_3$	5
**Figure S7.** HRESIMS spectrum of **1**	6
**Figure S8.** IR spectrum of **1**	7
**Figure S9.** ^{1}H NMR spectrum of **2** in $CDCl_3$	7
**Figure S10.** ^{13}C NMR spectrum of **2** in $CDCl_3$	8
**Figure S11.** HSQC spectrum of **2** in $CDCl_3$	8
**Figure S12.** HMBC spectrum of **2** in $CDCl_3$	9
**Figure S13.** ^{1}H−^{1}H COSY spectrum of **2** in $CDCl_3$	9
**Figure S14.** NOESY spectrum of **2** in $CDCl_3$	10
**Figure S15.** HRESIMS spectrum of **2**	11
**Figure S16.** IR spectrum of **2**	12
**NMR and ECD calculation method of compound 1**	
**Figure S17.** Structures of isomers **1a–1d**	13
**Table S1.** Conformers and Boltzmann distributions of the optimized **1a**	13
**Table S2.** Cartesian coordinates of optimized **1a**	14
**Table S3.** Conformers and Boltzmann distributions of the optimized **1b**	20
**Table S4.** Cartesian coordinates of optimized **1b**	20
**Table S5.** Conformers and Boltzmann distributions of the optimized **1c**	27
**Table S6.** Cartesian coordinates of optimized **1c**	27
**Table S7.** Conformers and Boltzmann distributions of the optimized **1d**	35
**Table S8.** Cartesian coordinates of optimized **1d**	35
**Table S9.** DP4+ analysis of compound **1**	41
**Figure S18.** Experimental and calculated ECD spectra of **1**	41
**NMR and ECD calculation method of compound 2**	
**Figure S19.** Optimized geometries of dominant conformers of conformer **2a** and **2b**	42
**Table S10.** Energy analysis of **2**	42
**Table S11.** Cartesian coordinates of optimized **2**	42
**Figure S20.** Linear correlation plots of predicted versus experimental ^{13}C NMR chemical shifts	45
**Table S12.** Parameters of the calculated ^{13}C NMR chemical shifts of **2**	45
**Figure S21.** Experimental and calculated ECD spectra of **2**	46
**Figure S22.** ^{1}H NMR spectrum of **3** in $CDCl_3$	47
**Figure S23.** HRESIMS spectrum of **3**	48
**Figure S24.** ^{1}H NMR spectrum of **4** in $CDCl_3$	49
**Figure S25.** HRESIMS spectrum of **4**	50
**Table S13.** NMR Data for compounds **1**− **2** in $CDCl_3$	51

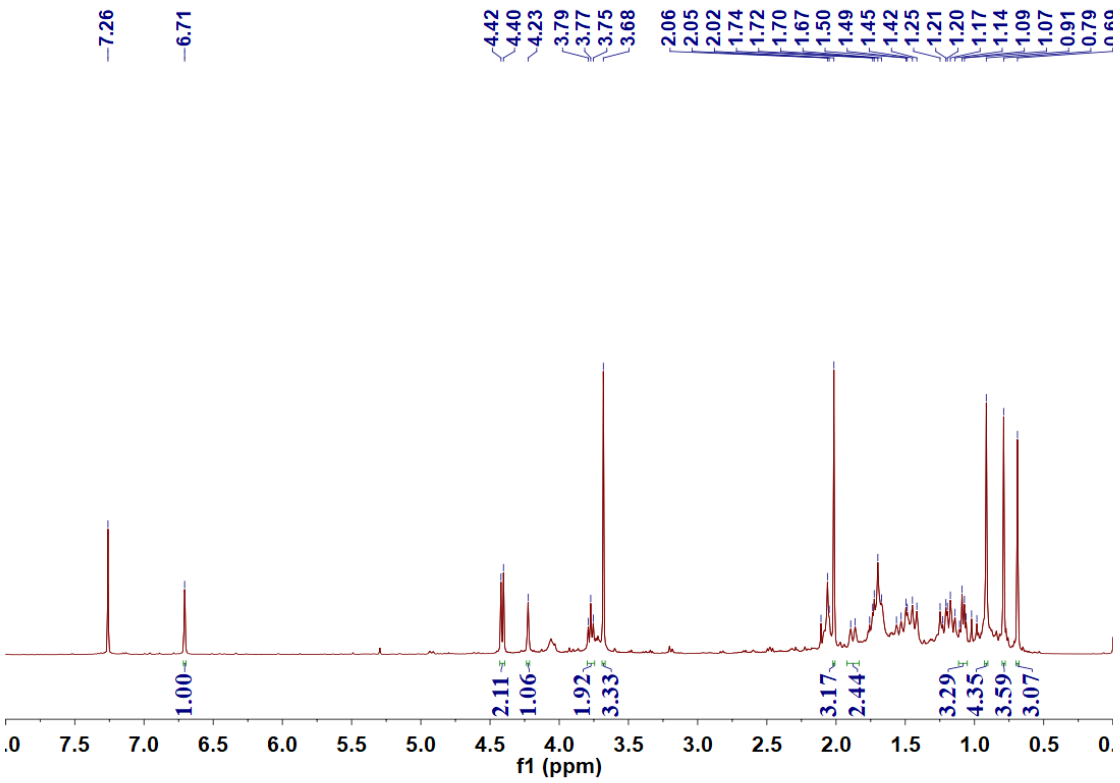

Figure S1. ^{1}H NMR spectrum of **1** in $CDCl_3$

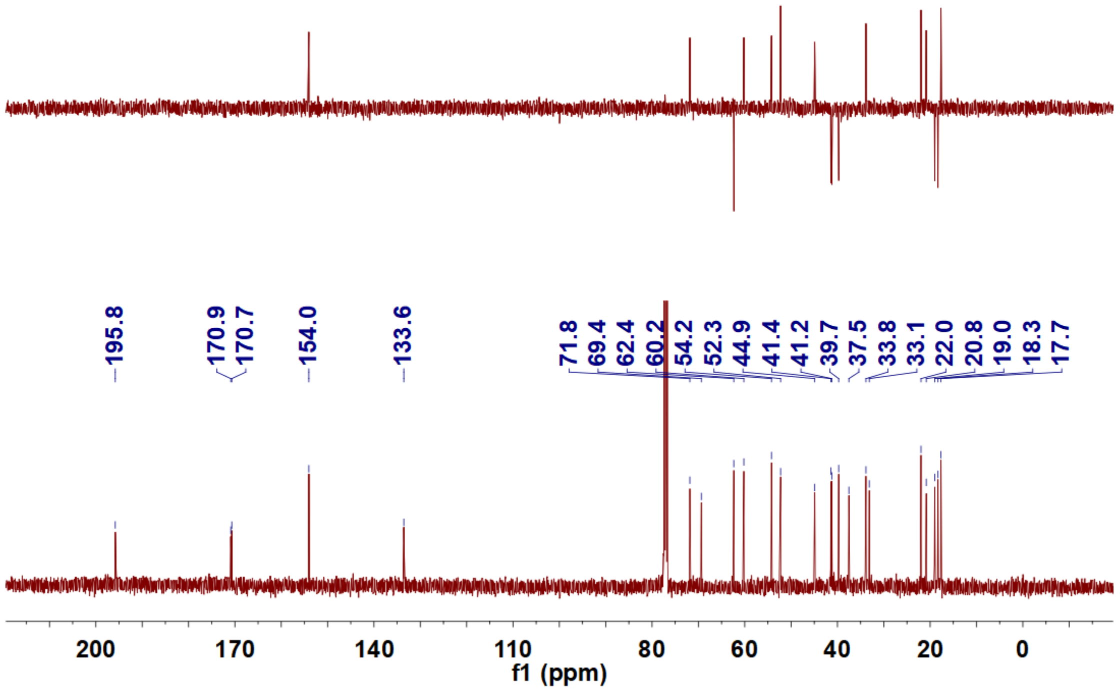

Figure S2. ^{13}C NMR and DEPT spectrum of **1** in $CDCl_3$

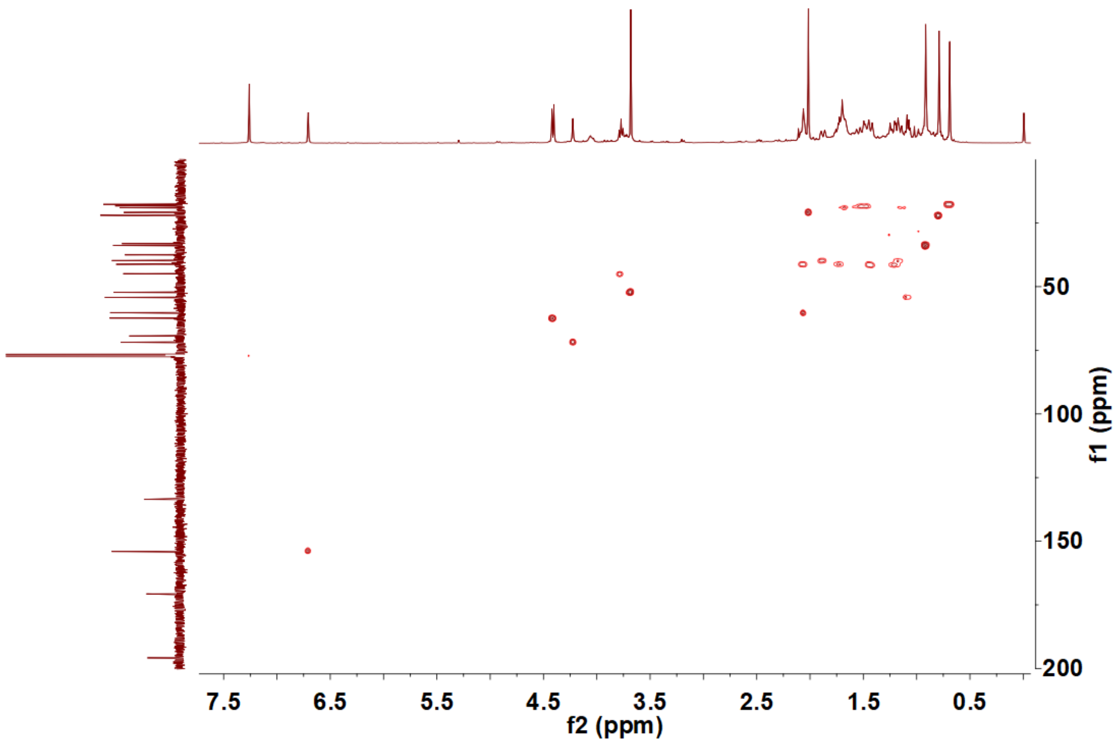

Figure S3. HSQC spectrum of **1** in $CDCl_3$

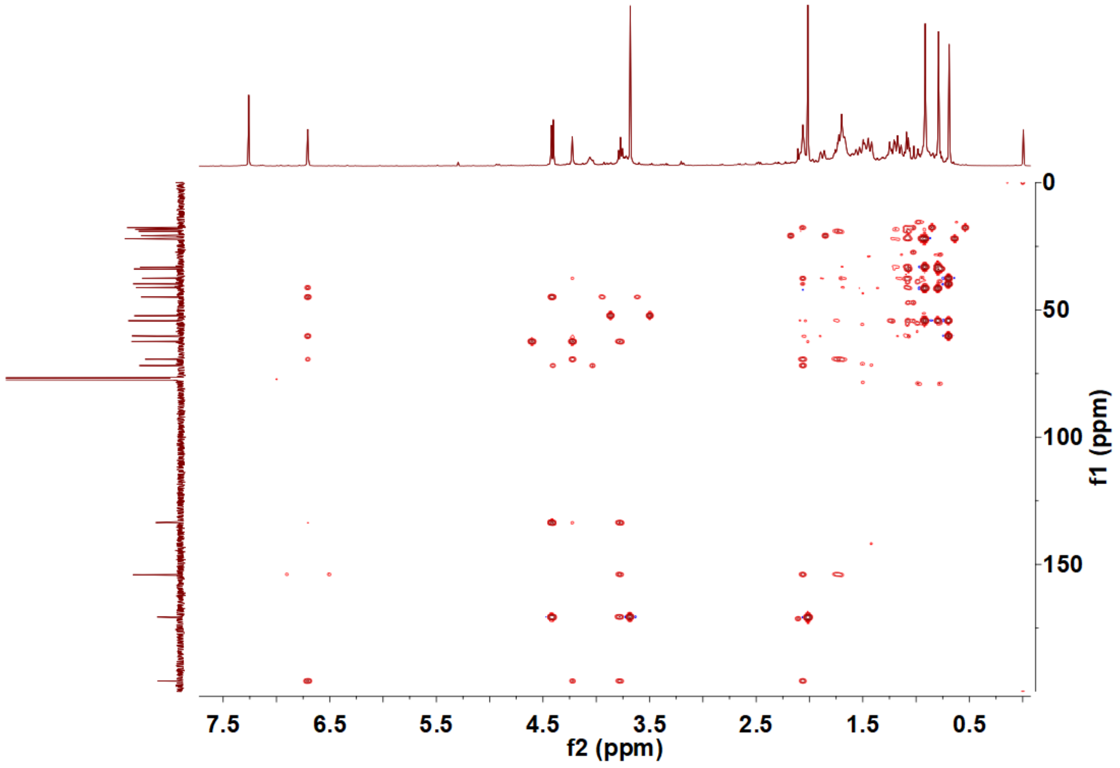

Figure S4. HMBC spectrum of **1** in $CDCl_3$

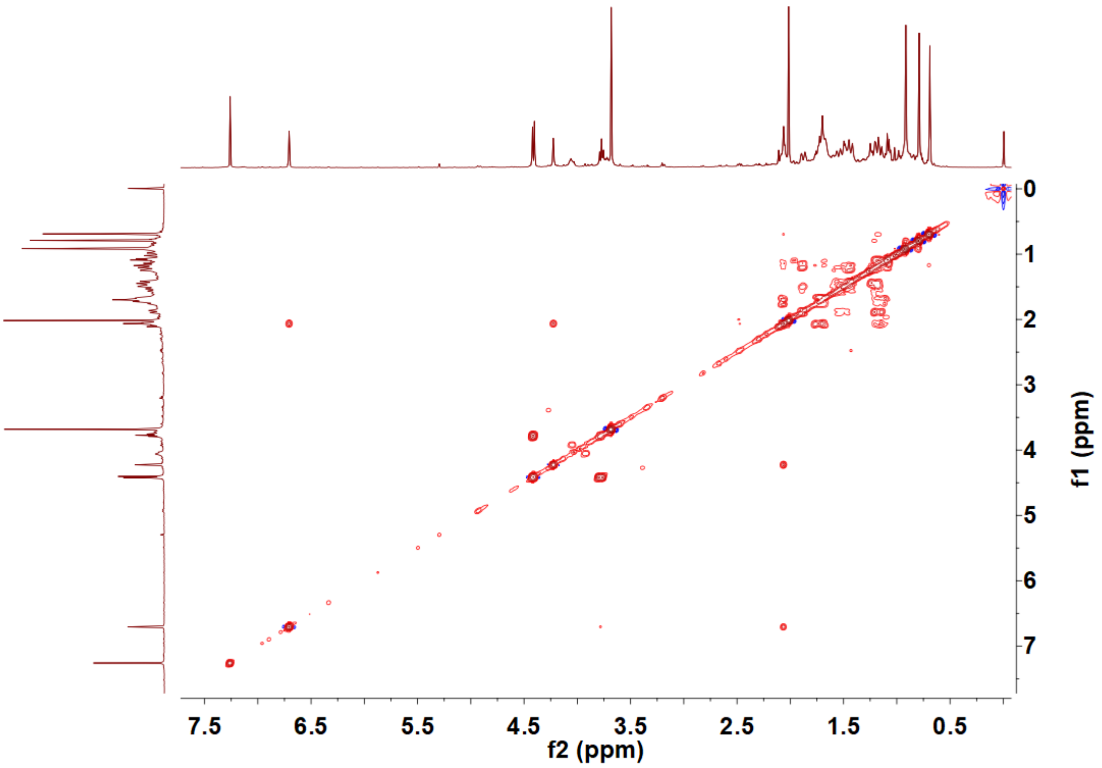

Figure S5. ^{1}H–^{1}H COSY spectrum of **1** in $CDCl_3$

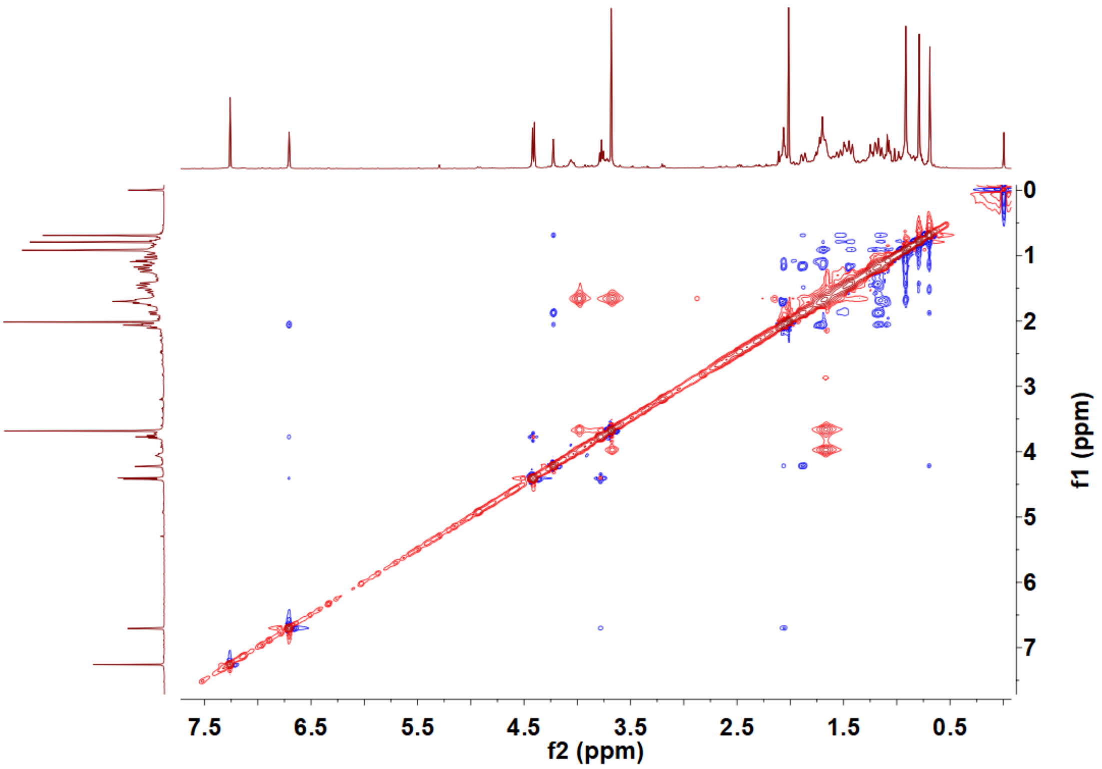

Figure S6. NOESY spectrum of **1** in $CDCl_3$

Elmt	Val.	Min	Max	Elmt	Val.	Min	Max	Elmt	Val.	Min	Max	Use Adduct
H	1	0	40	O	2	0	7	Cl	1	0	0	H
2H	1	0	0	F	1	0	0	Se	2	0	0	Na
B	3	0	0	Si	4	0	0	Br	1	0	0	K
C	4	0	30	P	3	0	0	I	3	0	0	N4
N	3	0	1	S	2	0	0					Cl

Error Margin (ppm):	20	DBE Range:	-1.0 - 1000.0	Electron Ions:	both
HC Ratio:	unlimited	Apply N Rule:	yes	Use MSn Info:	no
Max Isotopes:	all	Isotope RI (%):	1.00	Isotope Res:	10000
MSn Iso RI (%):	75.00	MSn Logic Mode:	AND	Max Results:	20

Event#: 1 MS(E+) Ret. Time : 1.253 Scan# : 189

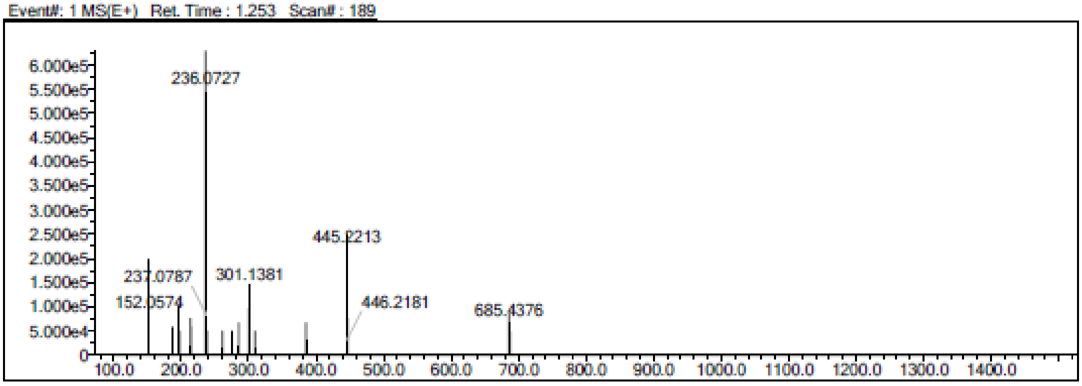

Measured region for 445.2213 m/z

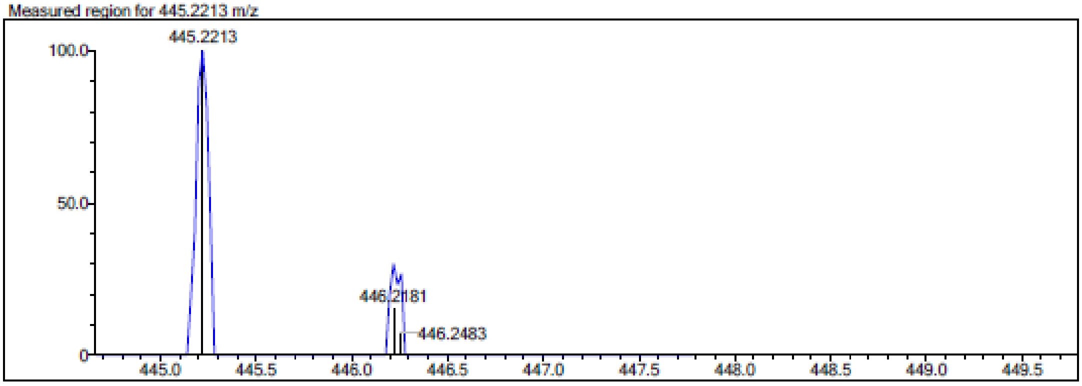

C23 H34 O7 [M+Na]+ : Predicted region for 445.2197 m/z

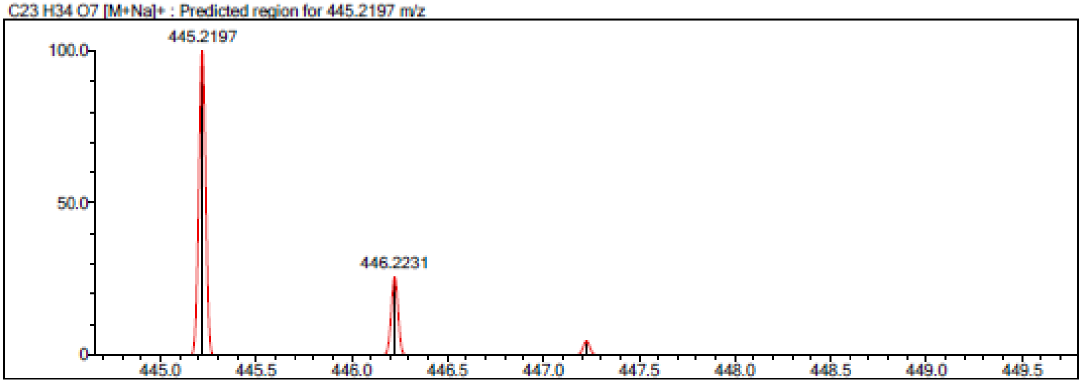

Rank	Score	Formula (M)	Ion	Meas. m/z	Pred. m/z	Df. (mDa)	Df. (ppm)	Iso	DBE
1	72.90	C23 H34 O7	[M+Na]+	445.2213	445.2197	1.6	3.59	77.95	7.0

Figure S7. HRESIMS spectrum of **1**

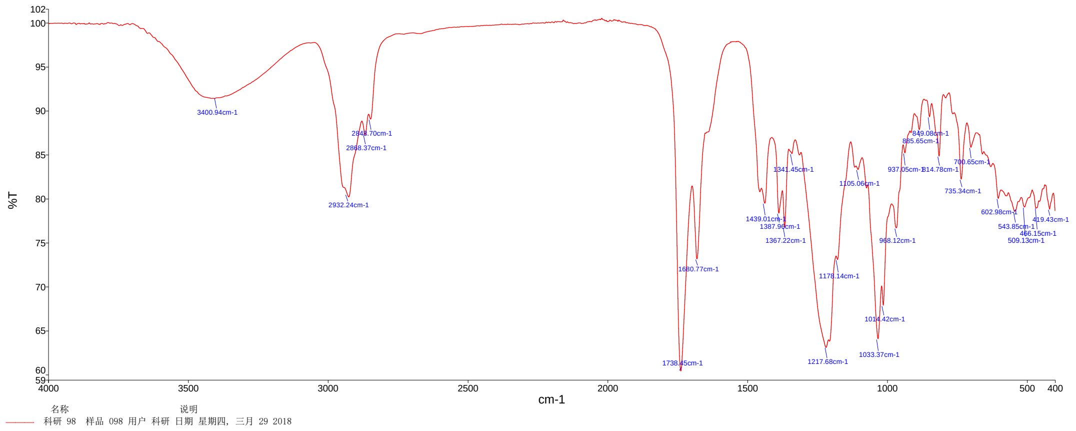

Figure S8. IR spectrum of **1**

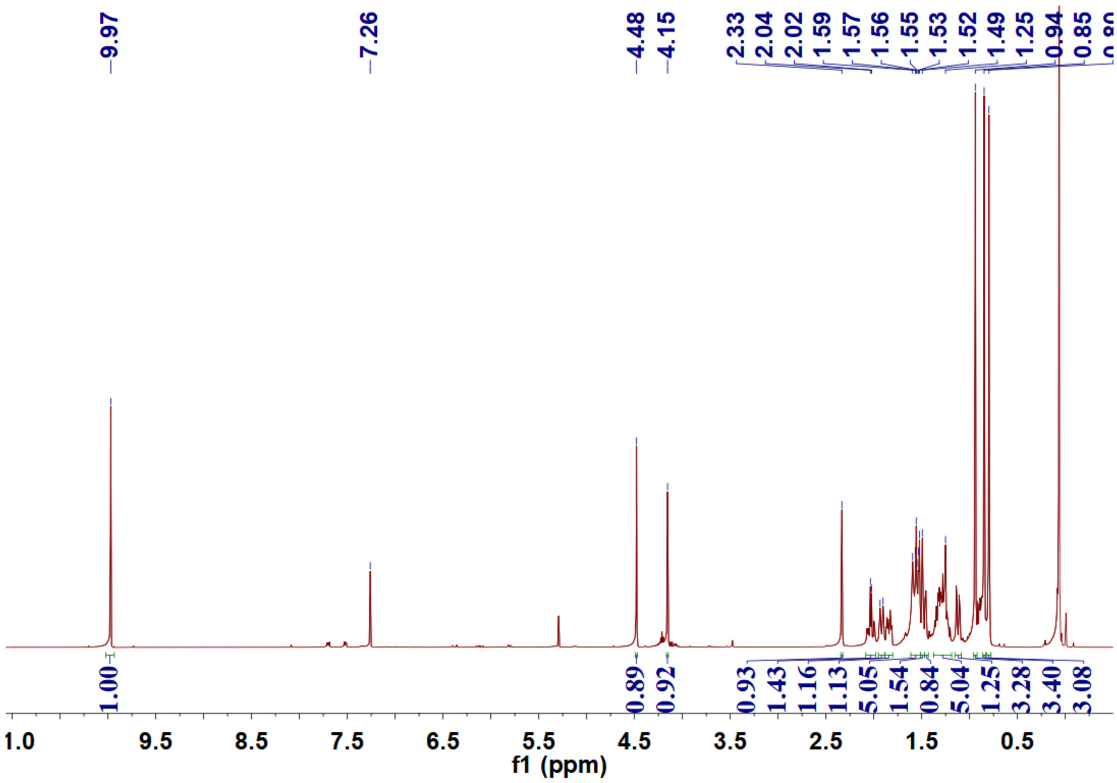

Figure S9. ^{1}H NMR spectrum of **2** in $CDCl_3$

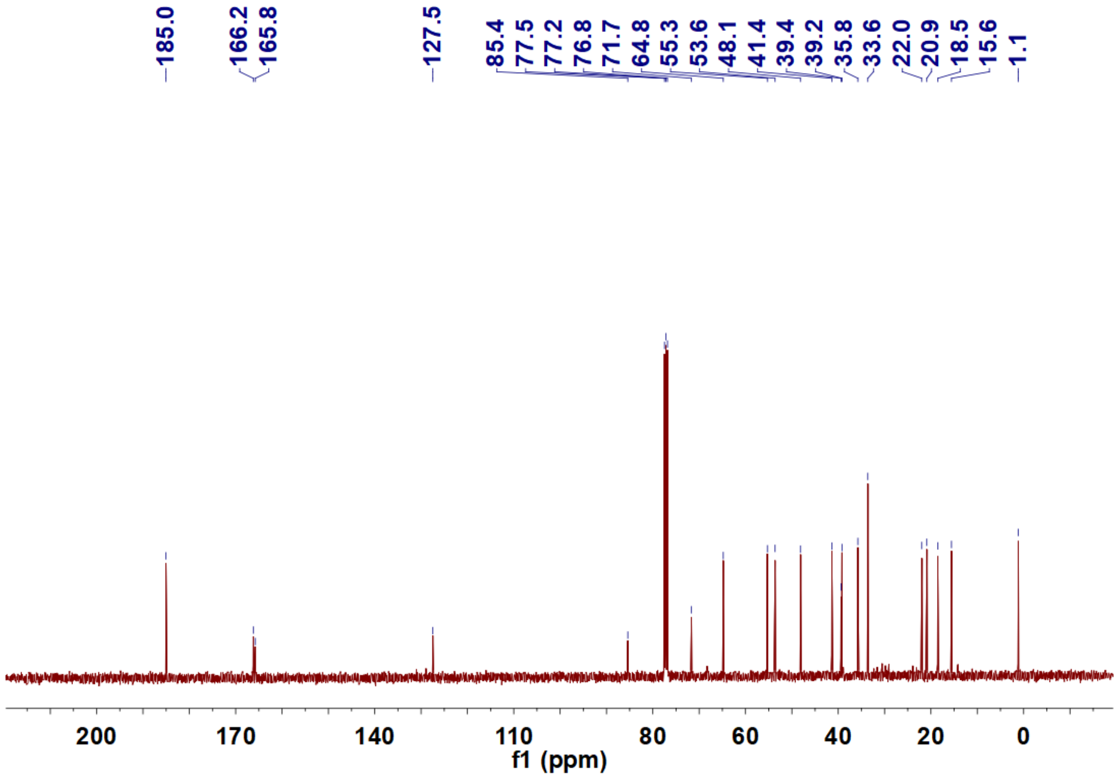

Figure S10. ^{13}C NMR spectrum of **2** in $CDCl_3$

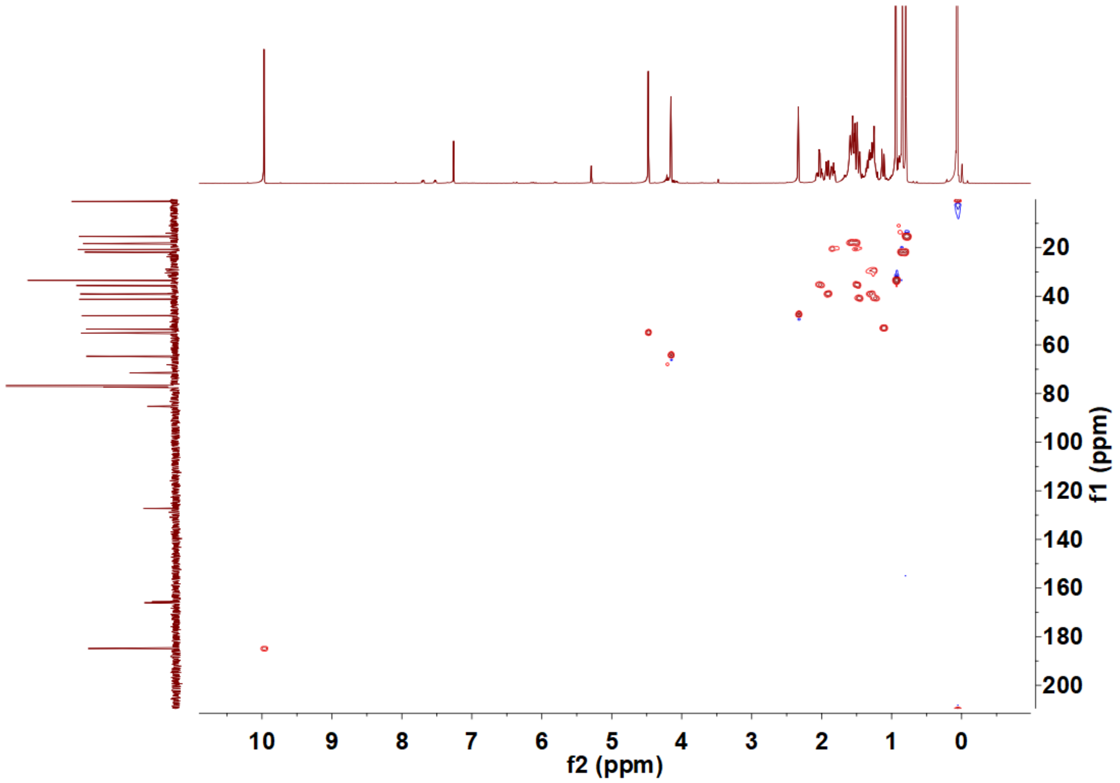

Figure S11. HSQC spectrum of **2** in $CDCl_3$

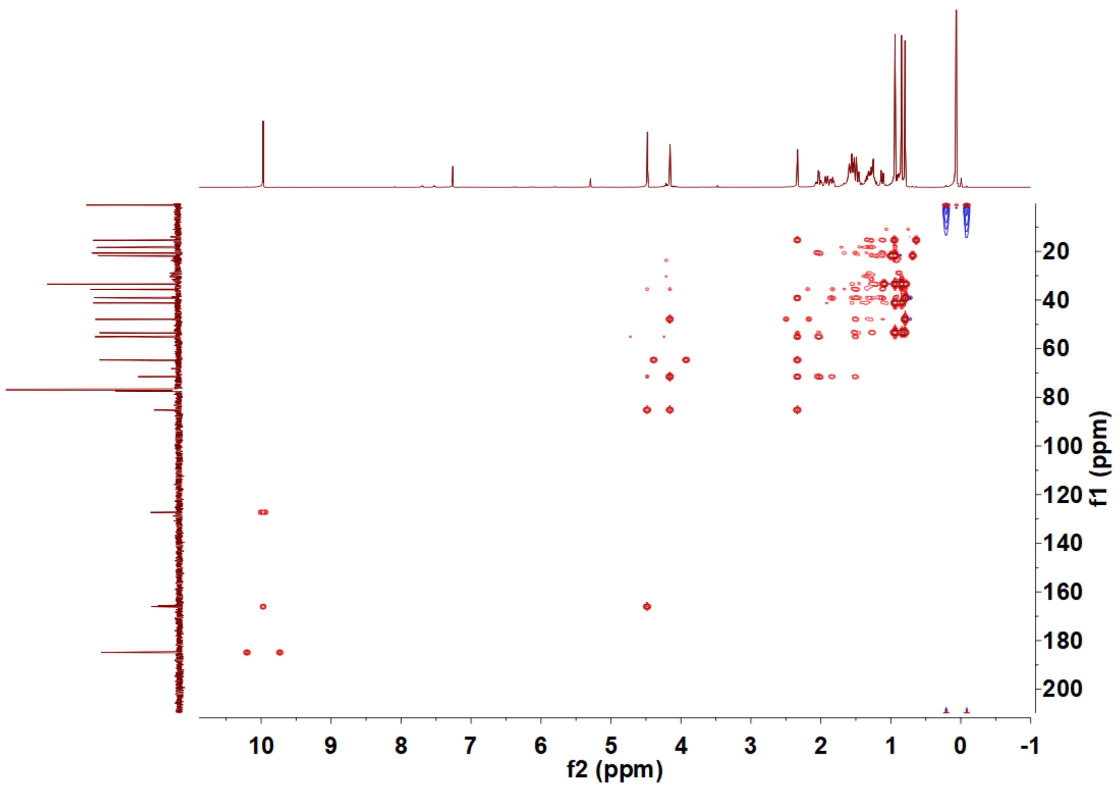

Figure S12. HMBC spectrum of **2** in $CDCl_3$

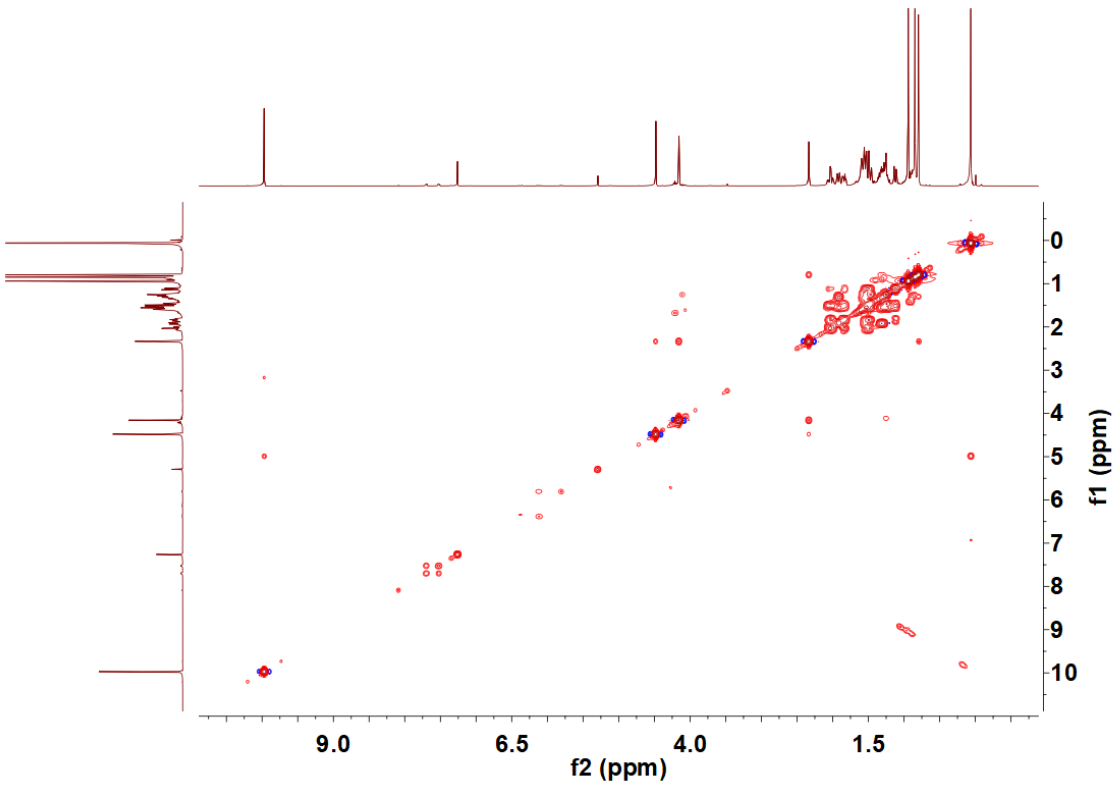

Figure S13. ^{1}H−^{1}H COSY spectrum of **2** in $CDCl_3$

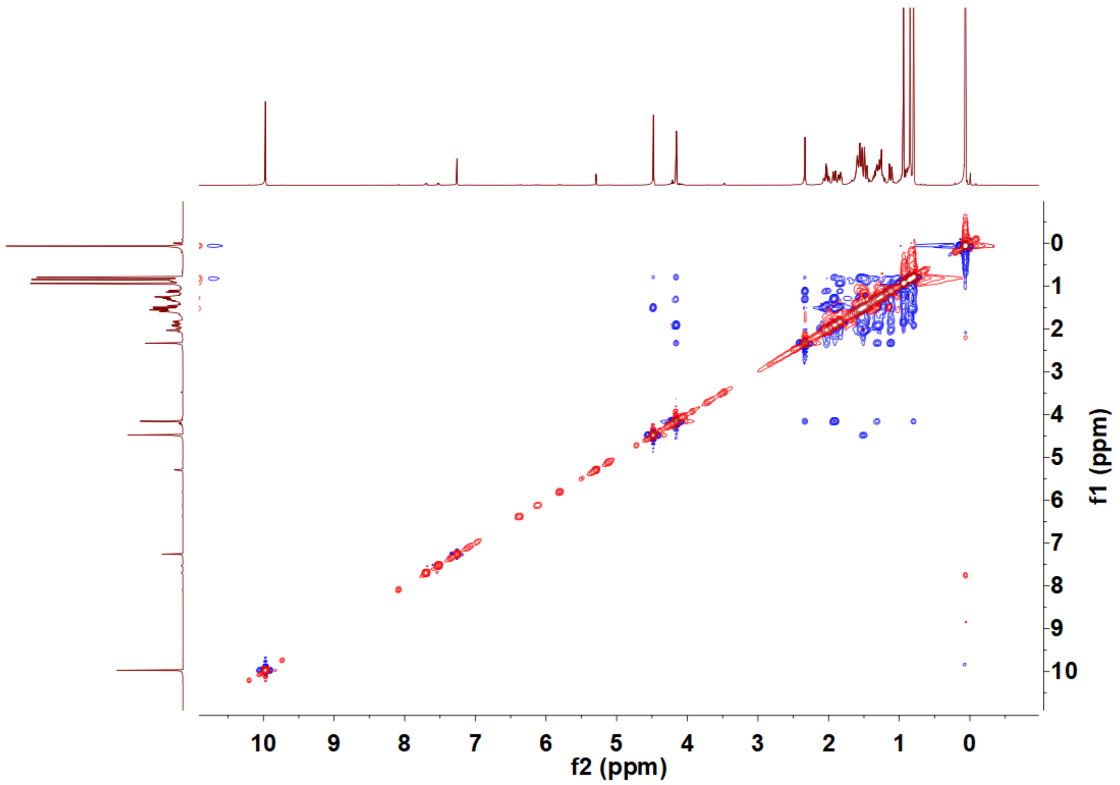

Figure S14. NOESY spectrum of **2** in $CDCl_3$

Elmt	Val.	Min	Max	Elmt	Val.	Min	Max	Elmt	Val.	Min	Max	Elmt	Val.	Min	Max	Use Adduct
H	1	0	24	N	3	0	0	P	3	0	0	K	1	0	0	H
B	3	0	0	O	2	0	5	S	2	0	0	Br	1	0	0	HCOO
C	4	0	20	F	1	0	0	Cl	1	0	0	I	3	0	0	CH3COO

Error Margin (ppm):	20	DBE Range:	not fixed	Electron Ions:	both
HC Ratio:	unlimited	Apply N Rule:	no	Use MSn Info:	no
Max Isotopes:	all	Isotope RI (%):	1.00	Isotope Res:	10000
MSn Iso RI (%):	75.00	MSn Logic Mode:	AND	Max Results:	100

Event#: 2 MS(E-) Ret. Time : 1.093 Scan# : 166

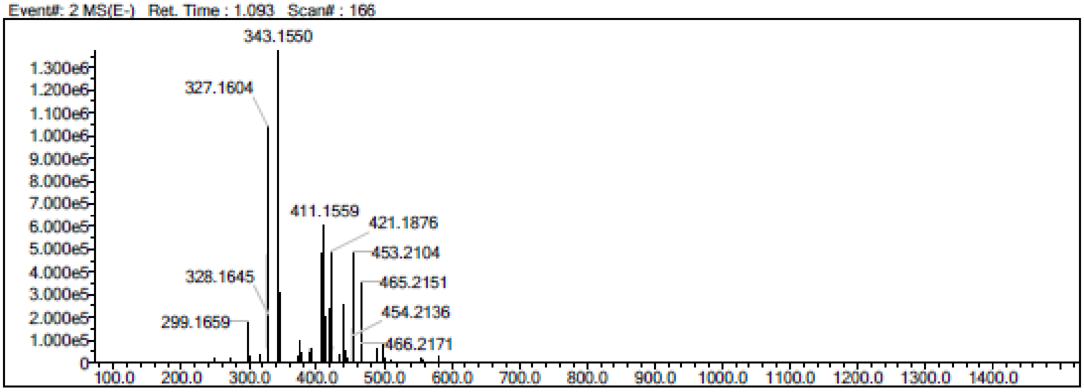

Measured region for 343.1550 m/z

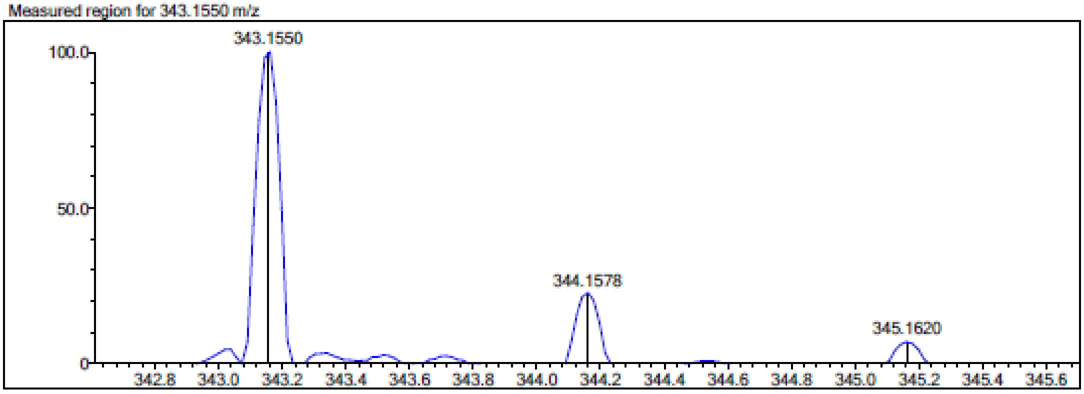

C20 H24 O5 [M-H]- : Predicted region for 343.1551 m/z

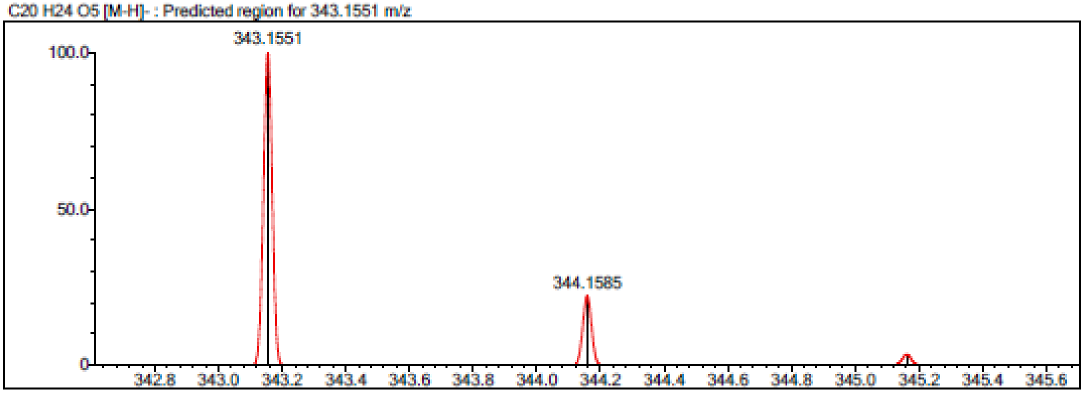

Rank	Score	Formula (M)	Ion	Meas. m/z	Pred. m/z	Df. (mDa)	Df. (ppm)	Iso	DBE
1	73.37	C20 H24 O5	[M-H]-	343.1550	343.1551	-0.1	-0.29	73.37	9.0

Figure S15. HRESIMS spectrum of **2**

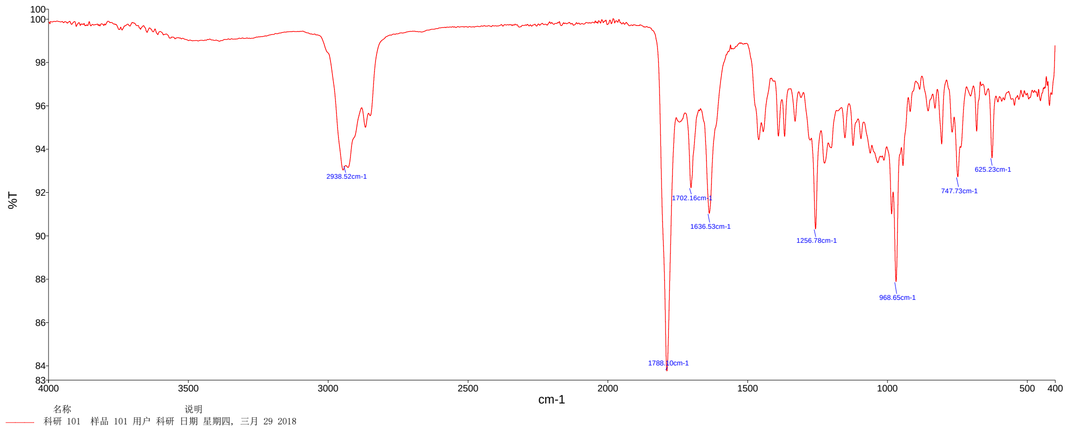

Figure S16. IR spectrum of **2**

NMR and ECD calculation method of compound 1

Calculation details

The random conformational searches were performed by SYBYL X 2.1.1 program using MMFF94s molecular force field. The obtained conformers were subsequently optimized by using Gaussion09 software at the B3LYP/6−31G(d) level in gas phase. The optimized stable conformers were selected for further NMR calculations at the mPW1PW91/6−311+G(d,p) level in chloroform and ECD calculations at the cam−B3LYP/6−31+G(d) level in acetonitrile. The overall theoretical NMR data were analyzed by using linear regression and DP4+ probability. The overall ECD data were weighted by Boltzmann distribution and produced by SpecDis 1.70.1 software.

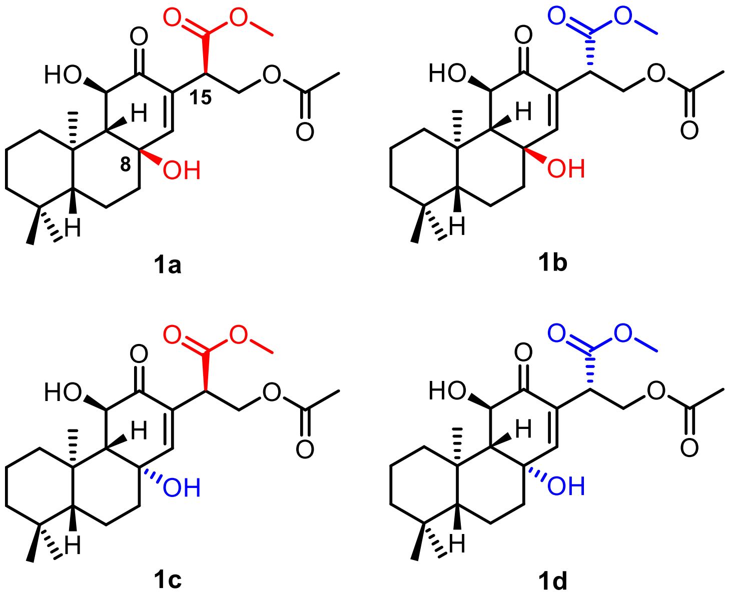

Figure S17. Structures of isomers **1a–1d**

Table S1. Conformers and Boltzmann distributions of the optimized **1a**

Compound	conformer	Energy (Hartree)	Energy (kcal/mol)	Population (%)
**1a**	**1a−1**	−1423.276303	−893119.357	13.03
**1a**	**1a−2**	−1423.277675	−893120.2178	55.7
**1a**	**1a−3**	−1423.277095	−893119.8539	30.14
**1a**	**1a−4**	−1423.274004	−893117.9143	1.14

Table S2. Cartesian coordinates of optimized **1a**

Conformer	**1a−1**				
Center	Atomic	Atomic	Coordinates (Angstroms)		
Number	Number	Type	X	Y	Z
1	6	0	−4.345374	1.533376	1.333837
2	1	0	−4.896174	1.822110	2.238229
3	1	0	−4.083907	2.472750	0.830843
4	6	0	−5.243453	0.678631	0.437150
5	1	0	−6.143724	1.239823	0.151486
6	1	0	−5.593610	−0.185137	1.022477
7	6	0	−4.545695	0.149960	−0.841679
8	6	0	−3.189436	−0.526991	−0.429417
9	6	0	−2.235996	0.280756	0.526993
10	6	0	−3.076952	0.771210	1.739642
11	1	0	−3.374205	−0.099506	2.342886
12	1	0	−2.469932	1.409682	2.392735
13	6	0	−2.393306	−1.130289	−1.601165
14	1	0	−3.062256	−1.635163	−2.305236
15	1	0	−1.884792	−0.346077	−2.175473
16	6	0	−1.388095	−2.156514	−1.073965
17	1	0	−0.802995	−2.602189	−1.888455
18	1	0	−1.931314	−2.979868	−0.595000
19	6	0	−0.425493	−1.601972	−0.018188
20	6	0	−1.158783	−0.747464	1.058464
21	1	0	−1.711927	−1.473555	1.668664
22	6	0	−5.477308	−0.926443	−1.448416
23	1	0	−5.544696	−1.810642	−0.802658
24	1	0	−5.148421	−1.255705	−2.439733
25	1	0	−6.490154	−0.521392	−1.563267
26	6	0	−4.414490	1.275319	−1.891452
27	1	0	−3.947588	2.182036	−1.501038
28	1	0	−5.410678	1.556878	−2.254494
29	1	0	−3.831861	0.950005	−2.760596
30	6	0	−1.570171	1.507872	−0.135572
31	1	0	−1.011631	1.268665	−1.041728
32	1	0	−0.861211	1.989036	0.546898
33	1	0	−2.305561	2.270317	−0.394854
34	6	0	0.740703	−0.862009	−0.645665
35	1	0	1.063066	−1.230631	−1.619523
36	6	0	1.474351	0.085445	−0.032624
37	6	0	1.106679	0.517456	1.326287
38	6	0	−0.118302	−0.118705	2.000556

39	1	0	−0.564560	0.645580	2.644596
40	8	0	0.406155	−1.181368	2.832868
41	1	0	1.117421	−0.791957	3.370623
42	8	0	1.811170	1.274419	1.988966
43	1	0	−3.511744	−1.386345	0.182928
44	8	0	0.158205	−2.777033	0.593102
45	1	0	0.545971	−2.484049	1.441932
46	6	0	2.751222	0.639411	−0.628644
47	1	0	2.805800	0.304922	−1.671206
48	6	0	4.016406	0.105482	0.086071
49	1	0	4.145229	0.585693	1.054119
50	1	0	4.896527	0.272976	−0.536733
51	6	0	2.705137	2.164845	−0.684403
52	8	0	1.697200	2.813930	−0.857824
53	8	0	3.935819	2.706906	−0.583212
54	6	0	3.985900	4.142603	−0.659202
55	1	0	3.404936	4.585529	0.153489
56	1	0	3.585981	4.488890	−1.615581
57	1	0	5.040564	4.402402	−0.565629
58	8	0	3.897984	−1.299009	0.392161
59	6	0	3.945621	−2.161349	−0.652814
60	6	0	3.666139	−3.571413	−0.197777
61	1	0	2.588122	−3.663511	−0.013384
62	1	0	4.188613	−3.795162	0.736347
63	1	0	3.960957	−4.273356	−0.979021
64	8	0	4.141457	−1.813889	−1.798902
Conformer	**1a−2**				
Center	Atomic	Atomic	Coordinates (Angstroms)		
Number	Number	Type	X	Y	Z
1	6	0	−4.125317	1.018796	1.951837
2	1	0	−4.574715	1.022851	2.953209
3	1	0	−3.902181	2.067720	1.720149
4	6	0	−5.125270	0.446935	0.944034
5	1	0	−6.043125	1.050813	0.929868
6	1	0	−5.423449	−0.555536	1.286357
7	6	0	−4.574786	0.324790	−0.499323
8	6	0	−3.188303	−0.413056	−0.448931
9	6	0	−2.131072	0.095143	0.599042
10	6	0	−2.833617	0.191658	1.982406
11	1	0	−3.079567	−0.823366	2.328596
12	1	0	−2.150167	0.616939	2.726817
13	6	0	−2.523711	−0.619530	−1.821967

14	1	0	−3.267512	−0.894929	−2.576588
15	1	0	−2.060467	0.309547	−2.176471
16	6	0	−1.491229	−1.745220	−1.732012
17	1	0	−0.999067	−1.918615	−2.697804
18	1	0	−2.002288	−2.680648	−1.474150
19	6	0	−0.418105	−1.536121	−0.656412
20	6	0	−1.014137	−1.019573	0.692822
21	1	0	−1.505668	−1.897578	1.132758
22	6	0	−5.575942	−0.552076	−1.289376
23	1	0	−5.579863	−1.586589	−0.924614
24	1	0	−5.359064	−0.572913	−2.362715
25	1	0	−6.591988	−0.155267	−1.173872
26	6	0	−4.550826	1.708191	−1.187108
27	1	0	−5.578869	2.045702	−1.368305
28	1	0	−4.045386	1.668996	−2.158393
29	1	0	−4.061906	2.481957	−0.591086
30	6	0	−1.531689	1.478555	0.256348
31	1	0	−0.802900	1.785877	1.013240
32	1	0	−2.297037	2.254923	0.242557
33	1	0	−1.017732	1.511426	−0.705868
34	6	0	0.726251	−0.674049	−1.157770
35	1	0	0.978951	−0.807076	−2.209935
36	6	0	1.499253	0.118398	−0.393736
37	6	0	1.194097	0.273393	1.044013
38	6	0	0.146746	−0.670889	1.649347
39	1	0	−0.209155	−0.204147	2.572886
40	8	0	0.802981	−1.915579	1.982174
41	1	0	1.756047	−1.766373	2.137780
42	8	0	1.807497	1.059566	1.755877
43	1	0	−3.452732	−1.422676	−0.091680
44	8	0	0.171291	−2.844629	−0.467393
45	1	0	0.581630	−2.810219	0.424072
46	6	0	2.724261	0.838023	−0.922555
47	1	0	2.735542	0.717055	−2.014224
48	6	0	4.034801	0.236697	−0.378490
49	1	0	4.086321	0.302429	0.707085
50	1	0	4.898673	0.729544	−0.823780
51	6	0	2.600043	2.345473	−0.680283
52	8	0	1.578004	2.971518	−0.851056
53	8	0	3.772452	2.903624	−0.321039
54	6	0	3.727568	4.319845	−0.070098
55	1	0	4.745439	4.599421	0.202387

56	1	0	3.035669	4.534288	0.747846
57	1	0	3.404376	4.857036	−0.965296
58	8	0	4.119303	−1.147465	−0.794449
59	6	0	3.785962	−2.097702	0.101484
60	6	0	3.730645	−3.460339	−0.536969
61	1	0	2.705157	−3.609956	−0.899283
62	1	0	3.950220	−4.225835	0.209625
63	1	0	4.414781	−3.536661	−1.384531
64	8	0	3.523257	−1.868560	1.269081
Conformer	**1a−3**				
Center	Atomic	Atomic	Coordinates (Angstroms)		
Number	Number	Type	X	Y	Z
1	6	0	−4.352255	1.239346	1.383404
2	1	0	−4.921521	1.474582	2.291801
3	1	0	−4.153214	2.203422	0.899101
4	6	0	−5.191886	0.343829	0.469883
5	1	0	−6.125029	0.851570	0.190044
6	1	0	−5.488712	−0.549096	1.040387
7	6	0	−4.459092	−0.119952	−0.814456
8	6	0	−3.061551	−0.712481	−0.408938
9	6	0	−2.164459	0.142985	0.559385
10	6	0	−3.036291	0.556245	1.778462
11	1	0	−3.274435	−0.343715	2.364419
12	1	0	−2.473446	1.221089	2.444535
13	6	0	−2.227494	−1.246917	−1.586684
14	1	0	−2.862497	−1.783571	−2.298462
15	1	0	−1.769829	−0.424401	−2.150167
16	6	0	−1.158620	−2.212340	−1.072513
17	1	0	−0.548192	−2.611542	−1.892326
18	1	0	−1.647054	−3.073791	−0.601170
19	6	0	−0.230621	−1.608934	−0.011781
20	6	0	−1.013956	−0.812478	1.075235
21	1	0	−1.510281	−1.579559	1.684040
22	6	0	−5.317203	−1.246268	−1.438750
23	1	0	−5.325613	−2.143199	−0.807255
24	1	0	−4.967975	−1.537280	−2.435035
25	1	0	−6.354650	−0.907559	−1.547852
26	6	0	−4.400440	1.026984	−1.847774
27	1	0	−5.410057	1.236746	−2.222073
28	1	0	−3.782239	0.761185	−2.712462
29	1	0	−4.011442	1.961730	−1.437496
30	6	0	−1.585967	1.422072	−0.087788

31	1	0	−2.372320	2.132529	−0.345109
32	1	0	−1.008286	1.228810	−0.993506
33	1	0	−0.918056	1.943257	0.607258
34	6	0	0.891302	−0.797547	−0.631649
35	1	0	1.246324	−1.153047	−1.598961
36	6	0	1.559939	0.198264	−0.018572
37	6	0	1.145018	0.624046	1.328818
38	6	0	−0.013155	−0.117399	2.013644
39	1	0	−0.509156	0.599351	2.675795
40	8	0	0.605911	−1.147466	2.820277
41	1	0	1.285446	−0.713508	3.364625
42	8	0	1.763491	1.469421	1.969523
43	1	0	−3.327349	−1.598980	0.191588
44	8	0	0.423696	−2.752516	0.586502
45	1	0	0.816405	−2.438710	1.425413
46	6	0	2.815078	0.810622	−0.604670
47	1	0	2.868662	0.509994	−1.657165
48	6	0	4.101465	0.304294	0.089227
49	1	0	4.214059	0.767471	1.068046
50	1	0	4.969601	0.536350	−0.529846
51	6	0	2.833791	2.335617	−0.594244
52	8	0	3.821201	3.008046	−0.395321
53	8	0	1.626236	2.851055	−0.908697
54	6	0	1.566297	4.287761	−0.942071
55	1	0	1.817427	4.700507	0.038212
56	1	0	0.537781	4.530403	−1.210377
57	1	0	2.262672	4.683542	−1.685852
58	8	0	4.051212	−1.111253	0.355005
59	6	0	4.124782	−1.941788	−0.713185
60	6	0	3.941841	−3.377693	−0.290190
61	1	0	2.877260	−3.539679	−0.078857
62	1	0	4.504048	−3.594097	0.622338
63	1	0	4.255197	−4.039558	−1.098717
64	8	0	4.271669	−1.554894	−1.854001
conformer	**1a−4**				
Center	Atomic	Atomic	Coordinates (Angstroms)		
Number	Number	Type	X	Y	Z
1	6	0	4.545889	−1.417735	−1.489097
2	1	0	5.144800	−2.303273	−1.737764
3	1	0	4.282721	−0.956102	−2.449363
4	6	0	5.380024	−0.456166	−0.639803
5	1	0	6.286040	−0.151803	−1.181687

6	1	0	5.724934	−0.997344	0.254137
7	6	0	4.613669	0.809569	−0.179696
8	6	0	3.248949	0.370586	0.466233
9	6	0	2.371087	−0.672164	−0.316911
10	6	0	3.281516	−1.857444	−0.740486
11	1	0	3.588957	−2.408254	0.160767
12	1	0	2.719224	−2.564943	−1.360896
13	6	0	2.370438	1.534370	0.958960
14	1	0	2.982079	2.312108	1.428029
15	1	0	1.855272	2.018992	0.120766
16	6	0	1.370692	1.022354	1.997064
17	1	0	0.717221	1.827617	2.356264
18	1	0	1.924910	0.664226	2.872941
19	6	0	0.502886	−0.162655	1.543742
20	6	0	1.297115	−1.220131	0.706506
21	1	0	1.863837	−1.786922	1.456705
22	6	0	5.478893	1.490924	0.907513
23	1	0	5.545232	0.875725	1.813199
24	1	0	5.093142	2.474829	1.195309
25	1	0	6.498872	1.641898	0.533211
26	6	0	4.490000	1.812865	−1.348520
27	1	0	5.477652	2.231149	−1.578812
28	1	0	3.832246	2.652080	−1.096679
29	1	0	4.115177	1.360939	−2.269916
30	6	0	1.706129	−0.081763	−1.582326
31	1	0	1.165265	−0.856270	−2.134146
32	1	0	2.444868	0.329577	−2.270490
33	1	0	0.993807	0.715725	−1.355718
34	6	0	−0.797903	0.281436	0.892178
35	1	0	−1.265257	1.134578	1.379683
36	6	0	−1.452091	−0.380646	−0.078016
37	6	0	−0.802757	−1.541022	−0.730083
38	6	0	0.296026	−2.218271	0.086410
39	1	0	0.790642	−2.954629	−0.553674
40	8	0	−0.371656	−2.912635	1.169640
41	1	0	−1.302965	−3.064807	0.918967
42	8	0	−1.194591	−2.003146	−1.793331
43	1	0	3.562038	−0.171601	1.374103
44	8	0	0.059654	−0.760435	2.779122
45	1	0	−0.317368	−1.626863	2.519820
46	6	0	−2.868010	−0.119507	−0.543126
47	1	0	−2.876679	−0.079183	−1.639846

48	6	0	−3.528069	1.157378	−0.019485
49	1	0	−3.529301	1.201509	1.074893
50	1	0	−4.563590	1.221019	−0.358021
51	6	0	−3.721974	−1.344369	−0.169688
52	8	0	−3.333024	−2.315775	0.450450
53	8	0	−4.978497	−1.218443	−0.622898
54	6	0	−5.860887	−2.324765	−0.347877
55	1	0	−5.950413	−2.481301	0.729702
56	1	0	−5.479646	−3.235952	−0.814809
57	1	0	−6.821098	−2.042015	−0.778638
58	8	0	−2.782494	2.273274	−0.543303
59	6	0	−3.286107	3.500257	−0.250379
60	6	0	−2.430011	4.587798	−0.854079
61	1	0	−1.412993	4.532644	−0.451755
62	1	0	−2.865941	5.560614	−0.625038
63	1	0	−2.359454	4.455188	−1.938492
64	8	0	−4.286548	3.667842	0.409448

Table S3. Conformers and Boltzmann distributions of the optimized **1b**

Compound	conformer	Energy (Hartree)	Energy (kcal/mol)	Population (%)
**1b**	**1b−1**	−1423.272546	−893116.9993	0.78
**1b**	**1b−2**	−1423.276172	−893119.275	36.12
**1b**	**1b−3**	−1423.276211	−893119.2995	37.65
**1b**	**1b−4**	−1423.275842	−893119.0677	25.46

Table S4. Cartesian coordinates of optimized **1b**

Conformer	**1b−1**				
Center	Atomic	Atomic	Coordinates (Angstroms)		
Number	Number	Type	X	Y	Z
1	6	0	4.558397	−0.500172	−2.084334
2	1	0	5.120518	−1.204131	−2.711302
3	1	0	4.186266	0.276045	−2.764842
4	6	0	5.489818	0.108001	−1.033078
5	1	0	6.326352	0.630486	−1.517233
6	1	0	5.935843	−0.713500	−0.452477
7	6	0	4.787365	1.077333	−0.048232
8	6	0	3.502085	0.377410	0.527053
9	6	0	2.535936	−0.329199	−0.492685
10	6	0	3.387555	−1.237144	−1.422030

11	1	0	3.793995	−2.069775	−0.828602
12	1	0	2.756468	−1.687388	−2.197622
13	6	0	2.689848	1.241082	1.509164
14	1	0	3.353588	1.810590	2.167759
15	1	0	2.086990	1.982401	0.971346
16	6	0	1.807116	0.345838	2.379097
17	1	0	1.201814	0.937284	3.077693
18	1	0	2.450389	−0.294068	2.994920
19	6	0	0.888210	−0.614209	1.607456
20	6	0	1.575833	−1.254980	0.356770
21	1	0	2.215179	−2.043630	0.775028
22	6	0	4.536877	2.442232	−0.728128
23	1	0	3.927360	3.101959	−0.100917
24	1	0	4.044485	2.359280	−1.700114
25	1	0	5.495262	2.948044	−0.898764
26	6	0	5.773909	1.329593	1.117065
27	1	0	5.938185	0.419674	1.707100
28	1	0	5.428045	2.115062	1.797712
29	1	0	6.745648	1.650051	0.721719
30	6	0	1.743987	0.668350	−1.369907
31	1	0	2.409527	1.321434	−1.934547
32	1	0	1.077165	1.312021	−0.789908
33	1	0	1.132202	0.137907	−2.105026
34	6	0	−0.475980	−0.013784	1.309967
35	1	0	−0.903756	0.555794	2.134793
36	6	0	−1.235732	−0.280685	0.231286
37	6	0	−0.668607	−1.083628	−0.874689
38	6	0	0.502306	−1.982632	−0.483160
39	1	0	0.919162	−2.414265	−1.397692
40	8	0	−0.057007	−3.047842	0.324687
41	1	0	−1.013253	−3.114754	0.135633
42	8	0	−1.158901	−1.121662	−1.996200
43	1	0	3.911830	−0.453032	1.125770
44	8	0	0.578404	−1.645413	2.567919
45	1	0	0.170167	−2.365571	2.043575
46	6	0	−2.698748	0.097287	0.134003
47	1	0	−2.985290	0.618307	1.054321
48	6	0	−2.980583	1.072789	−1.035909
49	1	0	−2.934820	0.558965	−1.993585
50	1	0	−2.226084	1.862631	−1.003732
51	6	0	−3.518856	−1.197131	0.077655
52	8	0	−3.055986	−2.323133	0.140471

53	8	0	−4.834607	−0.957036	−0.008002
54	6	0	−5.681700	−2.121423	−0.054426
55	1	0	−5.428022	−2.742492	−0.916725
56	1	0	−6.697386	−1.735939	−0.142013
57	1	0	−5.568799	−2.711139	0.858732
58	8	0	−4.287941	1.672250	−0.980177
59	6	0	−4.464146	2.659514	−0.071391
60	6	0	−5.871288	3.202502	−0.138787
61	1	0	−5.980826	4.020335	0.574079
62	1	0	−6.586473	2.406658	0.094452
63	1	0	−6.093655	3.554211	−1.151289
64	8	0	−3.597675	3.038945	0.688201
Conformer	**1b−2**				
Center	Atomic	Atomic	Coordinates (Angstroms)		
Number	Number	Type	X	Y	Z
1	6	0	4.073185	−0.942270	−2.142389
2	1	0	4.584701	−1.674527	−2.780136
3	1	0	3.717495	−0.154339	−2.818242
4	6	0	5.062698	−0.371071	−1.123535
5	1	0	5.903039	0.115967	−1.637033
6	1	0	5.494061	−1.209234	−0.556017
7	6	0	4.435107	0.625960	−0.116677
8	6	0	3.145743	−0.025462	0.502211
9	6	0	2.110714	−0.674248	−0.487440
10	6	0	2.889899	−1.622314	−1.442482
11	1	0	3.274229	−2.473462	−0.860864
12	1	0	2.215115	−2.042618	−2.198034
13	6	0	2.417046	0.850446	1.537893
14	1	0	3.133544	1.376629	2.176737
15	1	0	1.822192	1.629219	1.043300
16	6	0	1.534081	−0.027542	2.424822
17	1	0	1.002033	0.566608	3.178515
18	1	0	2.169088	−0.729759	2.978297
19	6	0	0.517174	−0.886771	1.661222
20	6	0	1.128589	−1.554025	0.386746
21	1	0	1.732796	−2.384155	0.775497
22	6	0	4.213901	2.000004	−0.787524
23	1	0	5.184423	2.468270	−0.992834
24	1	0	3.654387	2.682099	−0.137700
25	1	0	3.683007	1.937890	−1.740295
26	6	0	5.472832	0.839503	1.011809
27	1	0	5.187262	1.644402	1.697373

28	1	0	6.443205	1.113427	0.579910
29	1	0	5.617308	−0.073218	1.602732
30	6	0	1.339671	0.356511	−1.344248
31	1	0	0.657578	−0.151514	−2.034248
32	1	0	2.013512	0.949058	−1.963251
33	1	0	0.739620	1.052957	−0.755191
34	6	0	−0.768107	−0.130804	1.380756
35	1	0	−1.089350	0.533068	2.183536
36	6	0	−1.582828	−0.326220	0.327930
37	6	0	−1.186768	−1.275893	−0.729800
38	6	0	−0.002972	−2.202532	−0.431560
39	1	0	0.356102	−2.594658	−1.388399
40	8	0	−0.533632	−3.293040	0.357016
41	1	0	−1.381355	−3.564842	−0.035336
42	8	0	−1.832961	−1.424182	−1.762282
43	1	0	3.544194	−0.883930	1.068221
44	8	0	0.132369	−1.898241	2.612161
45	1	0	−0.381296	−2.559447	2.106953
46	6	0	−2.925880	0.355109	0.182789
47	1	0	−3.142464	0.905256	1.107652
48	6	0	−2.978178	1.380441	−0.968788
49	1	0	−3.941218	1.890444	−0.969738
50	1	0	−2.802791	0.892827	−1.926393
51	6	0	−4.025291	−0.705805	0.065441
52	8	0	−3.928100	−1.844717	0.468870
53	8	0	−5.144752	−0.206060	−0.492733
54	6	0	−6.246813	−1.125299	−0.598901
55	1	0	−7.053118	−0.559318	−1.065630
56	1	0	−6.545490	−1.478123	0.391420
57	1	0	−5.968001	−1.982853	−1.216150
58	8	0	−1.921257	2.358148	−0.848440
59	6	0	−2.134722	3.379801	0.016231
60	6	0	−0.977212	4.350120	−0.005925
61	1	0	−0.818492	4.725850	−1.021769
62	1	0	−0.056242	3.842995	0.301177
63	1	0	−1.185016	5.179146	0.671250
64	8	0	−3.126076	3.483598	0.705809
Conformer	**1b−3**				
Center	Atomic	Atomic	Coordinates (Angstroms)		
Number	Number	Type	X	Y	Z
1	6	0	4.020433	−1.741686	−1.720264
2	1	0	4.427732	−2.690409	−2.092848

3	1	0	3.812249	−1.136390	−2.611600
4	6	0	5.058715	−1.052603	−0.831451
5	1	0	5.980174	−0.858670	−1.397623
6	1	0	5.336051	−1.747707	−0.024637
7	6	0	4.568502	0.269841	−0.189333
8	6	0	3.176435	0.014345	0.495139
9	6	0	2.080471	−0.739535	−0.342555
10	6	0	2.725870	−2.018211	−0.945721
11	1	0	2.955170	−2.718296	−0.128631
12	1	0	2.013124	−2.531080	−1.602480
13	6	0	2.563550	1.246487	1.184957
14	1	0	3.335447	1.825338	1.702354
15	1	0	2.116933	1.926598	0.449131
16	6	0	1.525872	0.799247	2.215593
17	1	0	1.066027	1.657145	2.722471
18	1	0	2.027705	0.214530	2.996007
19	6	0	0.415768	−0.108355	1.663790
20	6	0	0.958231	−1.191192	0.675173
21	1	0	1.427392	−1.942485	1.323824
22	6	0	4.584416	1.410102	−1.232775
23	1	0	4.085446	1.149384	−2.169322
24	1	0	5.621984	1.662528	−1.484294
25	1	0	4.112838	2.320431	−0.846406
26	6	0	5.596332	0.648099	0.903925
27	1	0	5.418596	1.645622	1.319916
28	1	0	6.608279	0.651142	0.480650
29	1	0	5.585030	−0.070513	1.732573
30	6	0	1.500989	0.111218	−1.496607
31	1	0	2.278173	0.419048	−2.196200
32	1	0	0.995236	1.015980	−1.152081
33	1	0	0.774874	−0.465192	−2.079055
34	6	0	−0.738154	0.693564	1.088245
35	1	0	−0.981758	1.599269	1.644095
36	6	0	−1.527159	0.316834	0.063204
37	6	0	−1.236359	−0.943203	−0.653246
38	6	0	−0.240628	−1.901016	0.013515
39	1	0	0.078578	−2.616070	−0.751864
40	8	0	−0.944528	−2.607665	1.055482
41	1	0	−1.893272	−2.684315	0.827300
42	8	0	−1.826151	−1.273460	−1.676766
43	1	0	3.421458	−0.689292	1.308542
44	8	0	−0.138637	−0.736642	2.833931

45	1	0	−0.683553	−1.477727	2.494763
46	6	0	−2.750780	1.099173	−0.403344
47	1	0	−2.867713	0.902757	−1.474503
48	6	0	−4.060501	0.668399	0.299559
49	1	0	−4.779172	1.487743	0.290365
50	1	0	−3.881831	0.351386	1.327939
51	6	0	−2.531329	2.592722	−0.217783
52	8	0	−3.019356	3.266124	0.666230
53	8	0	−1.696183	3.081555	−1.156388
54	6	0	−1.387993	4.482125	−1.041021
55	1	0	−2.298782	5.081976	−1.113176
56	1	0	−0.716341	4.701080	−1.871173
57	1	0	−0.900153	4.690148	−0.084969
58	8	0	−4.710508	−0.395367	−0.434451
59	6	0	−4.403556	−1.666738	−0.131064
60	6	0	−5.048608	−2.632736	−1.088393
61	1	0	−4.380832	−2.738085	−1.951854
62	1	0	−6.012296	−2.261468	−1.443507
63	1	0	−5.160501	−3.606951	−0.609477
64	8	0	−3.667812	−1.985638	0.788028
Conformer	**1b−4**				
Center	Atomic	Atomic	Coordinates (Angstroms)		
Number	Number	Type	X	Y	Z
1	6	0	−4.438796	−1.010783	1.557891
2	1	0	−5.220538	−1.763738	1.721616
3	1	0	−3.923047	−0.901944	2.519908
4	6	0	−5.082862	0.314007	1.142214
5	1	0	−5.767635	0.668394	1.924935
6	1	0	−5.703727	0.130691	0.252212
7	6	0	−4.069030	1.437151	0.805566
8	6	0	−3.005735	0.869576	−0.202536
9	6	0	−2.333806	−0.507406	0.153368
10	6	0	−3.467221	−1.518429	0.484774
11	1	0	−4.038720	−1.725051	−0.432355
12	1	0	−3.042528	−2.476087	0.809281
13	6	0	−1.942898	1.887900	−0.653674
14	1	0	−2.397500	2.867115	−0.835371
15	1	0	−1.188829	2.042769	0.128093
16	6	0	−1.286445	1.413406	−1.950878
17	1	0	−0.527240	2.123792	−2.302274
18	1	0	−2.045087	1.356332	−2.740787
19	6	0	−0.655465	0.018921	−1.859049

20	6	0	−1.593074	−1.005097	−1.152346
21	1	0	−2.382318	−1.215552	−1.886117
22	6	0	−3.477983	2.038866	2.099733
23	1	0	−3.072592	1.288552	2.781620
24	1	0	−4.261797	2.579221	2.644906
25	1	0	−2.678917	2.756087	1.881811
26	6	0	−4.866327	2.565446	0.108042
27	1	0	−5.731584	2.846172	0.721136
28	1	0	−5.243340	2.245891	−0.871390
29	1	0	−4.269552	3.471975	−0.038771
30	6	0	−1.366527	−0.440087	1.355829
31	1	0	−1.892590	−0.207327	2.281939
32	1	0	−0.568203	0.293728	1.239480
33	1	0	−0.875191	−1.405280	1.520140
34	6	0	0.732072	0.070121	−1.240597
35	1	0	1.288945	0.985750	−1.425376
36	6	0	1.336352	−0.953779	−0.608524
37	6	0	0.596795	−2.216518	−0.418003
38	6	0	−0.836281	−2.328987	−0.960610
39	1	0	−1.377337	−3.012717	−0.298676
40	8	0	−0.707426	−2.946924	−2.263993
41	1	0	−0.190407	−3.761162	−2.137009
42	8	0	1.127197	−3.224069	0.041563
43	1	0	−3.604399	0.649225	−1.102676
44	8	0	−0.441187	−0.365950	−3.232680
45	1	0	−0.284486	−1.330190	−3.232046
46	6	0	2.772371	−0.965526	−0.122232
47	1	0	3.185915	−1.953280	−0.363745
48	6	0	3.716066	0.052843	−0.763800
49	1	0	3.624334	0.038248	−1.854453
50	1	0	4.751495	−0.169269	−0.500912
51	6	0	2.838008	−0.868654	1.407242
52	8	0	1.919572	−0.590698	2.143816
53	8	0	4.087579	−1.141640	1.838690
54	6	0	4.275911	−1.082416	3.263081
55	1	0	5.326153	−1.323444	3.428780
56	1	0	3.629921	−1.808780	3.762742
57	1	0	4.045661	−0.082137	3.639131
58	8	0	3.383498	1.371888	−0.276039
59	6	0	4.291849	2.348014	−0.541979
60	6	0	3.833857	3.672423	0.020436
61	1	0	4.633582	4.406181	−0.085158

62	1	0	3.551869	3.565919	1.072193
63	1	0	2.947782	4.019392	−0.522733
64	8	0	5.315383	2.154215	−1.156828

Table S5. Conformers and Boltzmann distributions of the optimized **1c**

Compound	conformer	Energy (Hartree)	Energy (kcal/mol)	Population (%)
**1c**	**1c−1**	−1423.277815	−893120.3061	26.81
**1c**	**1c−2**	−1423.277862	−893120.3353	28.16
**1c**	**1c−3**	−1423.276447	−893119.4474	6.29
**1c**	**1c−4**	−1423.27773	−893120.2527	24.5
**1c**	**1c−5**	−1423.277218	−893119.9313	14.24

Table S6. Cartesian coordinates of optimized **1c**

Conformer	**1c−1**				
Center	Atomic	Atomic	Coordinates (Angstroms)		
Number	Number	Type	X	Y	Z
1	6	0	4.577893	−0.921253	1.700955
2	1	0	4.924612	−1.093570	2.728401
3	1	0	4.899380	−1.801148	1.128862
4	6	0	5.233903	0.343880	1.146407
5	1	0	6.328119	0.241159	1.135921
6	1	0	5.010841	1.176204	1.831307
7	6	0	4.748191	0.738552	−0.270300
8	6	0	3.176476	0.733713	−0.286405
9	6	0	2.447500	−0.542009	0.292605
10	6	0	3.047668	−0.811243	1.702306
11	1	0	2.759402	0.012228	2.373291
12	1	0	2.608047	−1.714415	2.126676
13	6	0	2.567146	1.176026	−1.628596
14	1	0	3.108897	2.036024	−2.034978
15	1	0	2.637195	0.379321	−2.376665
16	6	0	1.101240	1.567990	−1.441582
17	1	0	0.649805	1.875116	−2.392612
18	1	0	1.033426	2.421518	−0.752463
19	6	0	0.274146	0.408184	−0.879198
20	6	0	0.919877	−0.156152	0.432389
21	1	0	0.882595	0.667508	1.161329
22	6	0	5.403347	−0.164255	−1.338662
23	1	0	6.481580	0.035915	−1.378043
24	1	0	4.998282	0.033787	−2.337292
25	1	0	5.280975	−1.230489	−1.137515

26	6	0	5.252239	2.179050	−0.528813
27	1	0	6.326551	2.241317	−0.315327
28	1	0	4.743692	2.905258	0.117648
29	1	0	5.112680	2.494870	−1.568101
30	6	0	2.594794	−1.821694	−0.561869
31	1	0	3.625546	−2.178269	−0.582427
32	1	0	2.253503	−1.685634	−1.588687
33	1	0	1.997730	−2.628175	−0.121027
34	6	0	−1.157560	0.842455	−0.655245
35	1	0	−1.552101	1.556231	−1.378307
36	6	0	−1.979244	0.297697	0.259007
37	6	0	−1.440134	−0.700583	1.201454
38	6	0	−0.030290	−1.255245	0.962194
39	1	0	−0.154125	−2.011722	0.166780
40	8	0	0.402399	−1.883982	2.147299
41	1	0	−0.415468	−2.029718	2.664074
42	8	0	−2.096692	−1.142682	2.141493
43	1	0	2.915536	1.528445	0.434366
44	8	0	0.206301	−0.560939	−1.947537
45	1	0	−0.584365	−1.120392	−1.834641
46	6	0	−3.478314	0.567985	0.307777
47	1	0	−3.845307	0.244213	1.285696
48	6	0	−4.227436	−0.211944	−0.792927
49	1	0	−3.756595	−0.070971	−1.766864
50	1	0	−5.269282	0.109912	−0.843974
51	6	0	−3.759236	2.052597	0.140943
52	8	0	−3.985751	2.594539	−0.922464
53	8	0	−3.699134	2.700152	1.318008
54	6	0	−3.896005	4.124933	1.253395
55	1	0	−4.880675	4.356005	0.839398
56	1	0	−3.128189	4.588788	0.628785
57	1	0	−3.818503	4.475089	2.282573
58	8	0	−4.295869	−1.619556	−0.477237
59	6	0	−3.340002	−2.447012	−0.944836
60	6	0	−3.591327	−3.862770	−0.490736
61	1	0	−4.638928	−4.138161	−0.639265
62	1	0	−3.380298	−3.932501	0.582586
63	1	0	−2.935079	−4.543292	−1.034160
64	8	0	−2.392342	−2.091766	−1.620426
Conformer	**1c−2**				
Center	Atomic	Atomic	Coordinates (Angstroms)		
Number	Number	Type	X	Y	Z

1	6	0	4.606327	0.596331	1.779056
2	1	0	4.979191	1.177647	2.632611
3	1	0	4.981370	−0.423984	1.929884
4	6	0	5.161634	1.183016	0.480808
5	1	0	6.260119	1.147415	0.477696
6	1	0	4.889661	2.248838	0.441166
7	6	0	4.628835	0.499884	−0.803331
8	6	0	3.061182	0.414723	−0.719126
9	6	0	2.436853	−0.167748	0.609486
10	6	0	3.072736	0.612364	1.795243
11	1	0	2.736223	1.659476	1.750536
12	1	0	2.701445	0.215151	2.740446
13	6	0	2.402733	−0.192882	−1.970440
14	1	0	2.882002	0.179469	−2.881152
15	1	0	2.508275	−1.282895	−1.981935
16	6	0	0.919107	0.169513	−2.013699
17	1	0	0.430161	−0.264496	−2.896878
18	1	0	0.806260	1.260786	−2.093949
19	6	0	0.180036	−0.319714	−0.762749
20	6	0	0.887335	0.144421	0.549133
21	1	0	0.802258	1.241048	0.557521
22	6	0	5.332715	−0.855652	−1.031040
23	1	0	6.393305	−0.686115	−1.255487
24	1	0	4.901556	−1.394215	−1.882458
25	1	0	5.289046	−1.518053	−0.164052
26	6	0	5.022158	1.414652	−1.988641
27	1	0	6.092465	1.649686	−1.937815
28	1	0	4.472923	2.364206	−1.963467
29	1	0	4.842784	0.945255	−2.961750
30	6	0	2.668275	−1.680822	0.827666
31	1	0	3.721679	−1.908110	0.997359
32	1	0	2.315200	−2.287334	−0.007416
33	1	0	2.131395	−2.009405	1.724169
34	6	0	−1.262921	0.144056	−0.792784
35	1	0	−1.704462	0.274435	−1.780376
36	6	0	−2.036771	0.275465	0.297569
37	6	0	−1.420614	0.128071	1.630480
38	6	0	0.021898	−0.385241	1.721399
39	1	0	−0.053582	−1.479137	1.603510
40	8	0	0.521534	−0.066953	3.000555
41	1	0	−0.274346	0.117705	3.538936
42	8	0	−2.025213	0.381551	2.668358

43	1	0	2.753193	1.474909	−0.706790
44	8	0	0.140402	−1.767796	−0.769221
45	1	0	−0.573179	−2.052924	−1.371062
46	6	0	−3.539360	0.525337	0.224420
47	1	0	−3.904561	0.607694	1.251136
48	6	0	−4.258446	−0.619625	−0.511086
49	1	0	−3.983078	−0.645960	−1.564686
50	1	0	−5.342543	−0.513899	−0.421770
51	6	0	−3.820968	1.833262	−0.504897
52	8	0	−3.990544	1.929314	−1.703708
53	8	0	−3.825167	2.876638	0.342158
54	6	0	−4.017804	4.168172	−0.264069
55	1	0	−3.983783	4.882128	0.558668
56	1	0	−4.983996	4.210842	−0.773168
57	1	0	−3.223975	4.373298	−0.986925
58	8	0	−3.935527	−1.884142	0.110077
59	6	0	−3.090144	−2.716112	−0.533941
60	6	0	−2.797601	−3.948542	0.283387
61	1	0	−2.809618	−4.825011	−0.369362
62	1	0	−3.503157	−4.071631	1.106111
63	1	0	−1.783420	−3.851677	0.688415
64	8	0	−2.599232	−2.487703	−1.625060
Conformer	**1c−3**				
Center	Atomic	Atomic	Coordinates (Angstroms)		
Number	Number	Type	X	Y	Z
1	6	0	−4.799994	−1.117013	−1.732227
2	1	0	−5.013510	−1.503238	−2.737485
3	1	0	−5.320686	−1.790865	−1.039808
4	6	0	−5.349129	0.304928	−1.605184
5	1	0	−6.436981	0.315211	−1.760839
6	1	0	−4.918182	0.915246	−2.413484
7	6	0	−5.023278	0.990647	−0.254859
8	6	0	−3.485299	0.836133	0.030067
9	6	0	−2.868001	−0.611935	−0.097265
10	6	0	−3.286384	−1.164498	−1.490041
11	1	0	−2.791368	−0.565956	−2.270239
12	1	0	−2.916366	−2.183053	−1.609486
13	6	0	−3.014301	1.546644	1.312408
14	1	0	−3.486582	2.529270	1.407003
15	1	0	−3.293715	0.976151	2.204901
16	6	0	−1.497251	1.735659	1.284973
17	1	0	−1.143566	2.236855	2.198570

18	1	0	−1.218221	2.388991	0.445921
19	6	0	−0.761363	0.397660	1.145251
20	6	0	−1.298568	−0.430846	−0.061850
21	1	0	−1.048808	0.161075	−0.954646
22	6	0	−5.939288	0.448627	0.863797
23	1	0	−5.932981	−0.640554	0.936979
24	1	0	−6.975247	0.754349	0.671122
25	1	0	−5.658374	0.849340	1.844223
26	6	0	−5.358295	2.492511	−0.423621
27	1	0	−6.372932	2.605171	−0.824983
28	1	0	−4.669114	2.982849	−1.122548
29	1	0	−5.325921	3.040548	0.524265
30	6	0	−3.316771	−1.612691	0.992157
31	1	0	−4.370137	−1.876623	0.888266
32	1	0	−3.154363	−1.238371	2.003662
33	1	0	−2.750464	−2.544675	0.892263
34	6	0	0.734816	0.632058	1.022586
35	1	0	1.128101	1.507741	1.541875
36	6	0	1.582295	−0.179156	0.368509
37	6	0	1.030591	−1.347391	−0.344629
38	6	0	−0.433595	−1.721707	−0.117157
39	1	0	−0.473966	−2.193708	0.877436
40	8	0	−0.817714	−2.643941	−1.110328
41	1	0	0.022297	−2.952427	−1.506096
42	8	0	1.713736	−2.022925	−1.111303
43	1	0	−3.018795	1.391115	−0.802680
44	8	0	−0.972464	−0.388853	2.342484
45	1	0	−0.536800	0.072060	3.078163
46	6	0	3.073603	0.084385	0.259660
47	1	0	3.314253	0.868802	0.985929
48	6	0	3.444847	0.625648	−1.135574
49	1	0	2.687591	1.347744	−1.449589
50	1	0	3.520437	−0.181820	−1.861194
51	6	0	3.847278	−1.160225	0.704884
52	8	0	3.532247	−1.824990	1.668443
53	8	0	4.926644	−1.407439	−0.056499
54	6	0	5.687428	−2.569419	0.315891
55	1	0	5.069737	−3.467494	0.236747
56	1	0	6.052740	−2.476032	1.341813
57	1	0	6.518731	−2.610185	−0.388209
58	8	0	4.733140	1.273438	−1.148569
59	6	0	4.777858	2.529204	−0.646294

60	6	0	6.168525	3.105132	−0.767651
61	1	0	6.891881	2.451274	−0.270067
62	1	0	6.191366	4.098845	−0.319328
63	1	0	6.457293	3.166389	−1.822205
64	8	0	3.819197	3.099690	−0.167884
Conformer	**1c−4**				
Center	Atomic	Atomic	Coordinates (Angstroms)		
Number	Number	Type	X	Y	Z
1	6	0	−4.470117	−1.060610	−1.881282
2	1	0	−4.675614	−1.226141	−2.946960
3	1	0	−4.939091	−1.901994	−1.355274
4	6	0	−5.100193	0.261226	−1.439205
5	1	0	−6.188802	0.240895	−1.588728
6	1	0	−4.716146	1.060978	−2.090773
7	6	0	−4.796923	0.647229	0.029714
8	6	0	−3.248803	0.528650	0.270991
9	6	0	−2.546650	−0.814063	−0.178050
10	6	0	−2.952306	−1.067553	−1.658244
11	1	0	−2.505931	−0.281647	−2.286776
12	1	0	−2.523453	−2.009485	−2.001165
13	6	0	−2.804313	0.964631	1.678322
14	1	0	−3.338053	1.867797	1.989787
15	1	0	−3.032051	0.192265	2.420580
16	6	0	−1.303259	1.252583	1.691316
17	1	0	−0.966822	1.560666	2.692847
18	1	0	−1.079869	2.092472	1.017345
19	6	0	−0.488475	0.028887	1.255808
20	6	0	−0.991078	−0.546129	−0.104459
21	1	0	−0.790491	0.242015	−0.844798
22	6	0	−5.660765	−0.186320	1.001056
23	1	0	−5.569123	−1.262883	0.845726
24	1	0	−6.718639	0.072472	0.867536
25	1	0	−5.404473	0.020852	2.046235
26	6	0	−5.227140	2.124681	0.199204
27	1	0	−6.253011	2.257248	−0.165931
28	1	0	−4.581861	2.800891	−0.375637
29	1	0	−5.214650	2.451128	1.244503
30	6	0	−2.919531	−2.058718	0.659610
31	1	0	−2.285048	−2.902433	0.369055
32	1	0	−3.950253	−2.368938	0.480215
33	1	0	−2.788064	−1.903985	1.731335
34	6	0	0.986432	0.384894	1.180337

35	1	0	1.331679	1.143562	1.885691
36	6	0	1.872907	−0.194674	0.354985
37	6	0	1.388469	−1.227268	−0.584404
38	6	0	−0.047128	−1.733783	−0.438757
39	1	0	−0.039302	−2.409379	0.431528
40	8	0	−0.390306	−2.444578	−1.605648
41	1	0	0.462462	−2.619057	−2.052691
42	8	0	2.102356	−1.695645	−1.467717
43	1	0	−2.829759	1.282964	−0.418205
44	8	0	−0.633811	−1.014243	2.249211
45	1	0	−0.193324	−0.712422	3.060520
46	6	0	3.337060	0.187048	0.311189
47	1	0	3.533767	0.881658	1.137178
48	6	0	3.744251	0.912731	−0.989437
49	1	0	3.673591	0.242631	−1.844049
50	1	0	4.759348	1.298800	−0.892486
51	6	0	4.204410	−1.043925	0.586613
52	8	0	3.860385	−1.977791	1.275600
53	8	0	5.420772	−0.940032	0.015486
54	6	0	6.310197	−2.046288	0.254414
55	1	0	5.875115	−2.971879	−0.130096
56	1	0	6.501377	−2.157393	1.324710
57	1	0	7.229119	−1.800445	−0.277983
58	8	0	2.846447	2.000034	−1.299398
59	6	0	2.992024	3.136927	−0.579805
60	6	0	2.030946	4.199010	−1.059315
61	1	0	2.229295	4.437269	−2.109503
62	1	0	2.143488	5.095116	−0.448334
63	1	0	1.001727	3.830291	−0.999084
64	8	0	3.792004	3.267395	0.322476
Conformer	**1c−5**				
Center	Atomic	Atomic	Coordinates (Angstroms)		
Number	Number	Type	X	Y	Z
1	6	0	4.928404	−1.506604	0.760642
2	1	0	5.274148	−2.380612	1.328022
3	1	0	5.514898	−1.505381	−0.167212
4	6	0	5.201620	−0.235384	1.565829
5	1	0	6.277104	−0.124679	1.763497
6	1	0	4.718404	−0.340071	2.549208
7	6	0	4.671656	1.059651	0.901398
8	6	0	3.172905	0.834514	0.485082
9	6	0	2.835339	−0.470828	−0.336562

10	6	0	3.434934	−1.670889	0.451116
11	1	0	2.892429	−1.775548	1.402924
12	1	0	3.262559	−2.596182	−0.099080
13	6	0	2.502035	2.079697	−0.121351
14	1	0	2.784155	2.980205	0.432858
15	1	0	2.822882	2.234963	−1.157252
16	6	0	0.980983	1.932896	−0.073881
17	1	0	0.483401	2.815214	−0.506425
18	1	0	0.645574	1.871358	0.970935
19	6	0	0.503526	0.684294	−0.824369
20	6	0	1.259735	−0.593751	−0.348998
21	1	0	0.960751	−0.731675	0.699958
22	6	0	5.598133	1.495414	−0.254392
23	1	0	5.786289	0.701496	−0.979987
24	1	0	6.571062	1.801915	0.149710
25	1	0	5.186729	2.353101	−0.798513
26	6	0	4.738154	2.171836	1.976042
27	1	0	5.739910	2.197653	2.422159
28	1	0	4.020053	1.993720	2.786325
29	1	0	4.544649	3.168852	1.565462
30	6	0	3.387030	−0.498007	−1.780561
31	1	0	3.095792	0.377843	−2.361759
32	1	0	3.003561	−1.380769	−2.302786
33	1	0	4.475308	−0.576172	−1.796876
34	6	0	−0.998902	0.522904	−0.656676
35	1	0	−1.567210	1.441851	−0.521797
36	6	0	−1.642919	−0.655192	−0.686987
37	6	0	−0.846233	−1.890353	−0.843028
38	6	0	0.648019	−1.773563	−1.150380
39	1	0	0.717706	−1.522151	−2.221371
40	8	0	1.250508	−3.024450	−0.910196
41	1	0	0.503616	−3.654135	−0.851609
42	8	0	−1.352663	−3.005751	−0.754785
43	1	0	2.666096	0.668431	1.451695
44	8	0	0.762953	0.851209	−2.240239
45	1	0	0.291673	1.650227	−2.528176
46	6	0	−3.134329	−0.855676	−0.517669
47	1	0	−3.420884	−1.719654	−1.130465
48	6	0	−4.031247	0.296807	−0.973264
49	1	0	−5.073935	−0.024058	−1.007784
50	1	0	−3.744302	0.645271	−1.970383
51	6	0	−3.458287	−1.260030	0.928069

52	8	0	−2.692456	−1.218780	1.861908
53	8	0	−4.738514	−1.678100	1.018518
54	6	0	−5.160231	−2.091739	2.329988
55	1	0	−4.553071	−2.931590	2.677034
56	1	0	−6.203124	−2.389986	2.220567
57	1	0	−5.067234	−1.266370	3.040650
58	8	0	−3.912130	1.387748	−0.032694
59	6	0	−4.788279	2.413268	−0.206012
60	6	0	−4.591512	3.471552	0.852851
61	1	0	−4.662775	3.027973	1.850844
62	1	0	−5.346856	4.248625	0.732333
63	1	0	−3.592728	3.912416	0.763302
64	8	0	−5.601428	2.446000	−1.100927

Table S7. Conformers and Boltzmann distributions of the optimized **1d**

Compound	conformer	Energy (Hartree)	Energy (kcal/mol)	Population (%)
**1d**	**1d−1**	−1423.279074	−893121.0959	87.6
**1d**	**1d−2**	−1423.276722	−893119.6202	7.26
**1d**	**1d−3**	−1423.276396	−893119.4157	5.14

Table S8. Cartesian coordinates of optimized **1d**

Conformer	**1d−1**				
Center	Atomic	Atomic	Coordinates (Angstroms)		
Number	Number	Type	X	Y	Z
1	6	0	−4.398108	−1.597960	1.450925
2	1	0	−4.664064	−2.561783	1.904404
3	1	0	−4.736829	−0.833424	2.162116
4	6	0	−5.126951	−1.443097	0.115317
5	1	0	−6.215767	−1.475834	0.261290
6	1	0	−4.876152	−2.308497	−0.517055
7	6	0	−4.756857	−0.153977	−0.658424
8	6	0	−3.190684	−0.044871	−0.736262
9	6	0	−2.383955	−0.215411	0.611024
10	6	0	−2.875348	−1.533020	1.276302
11	1	0	−2.560244	−2.382674	0.652279
12	1	0	−2.382612	−1.668349	2.239676
13	6	0	−2.691890	1.161918	−1.550759
14	1	0	−3.282441	1.279952	−2.464783
15	1	0	−2.799554	2.093962	−0.986265

16	6	0	−1.226157	0.972130	−1.942115
17	1	0	−0.854111	1.836396	−2.506479
18	1	0	−1.127439	0.090585	−2.590586
19	6	0	−0.327034	0.783391	−0.718086
20	6	0	−0.862471	−0.364218	0.202310
21	1	0	−0.801817	−1.278703	−0.406335
22	6	0	−5.455701	1.072506	−0.031413
23	1	0	−5.290033	1.160662	1.044361
24	1	0	−6.539410	0.997552	−0.186222
25	1	0	−5.124248	2.007635	−0.496420
26	6	0	−5.324429	−0.313490	−2.089594
27	1	0	−6.381874	−0.600983	−2.039927
28	1	0	−4.794728	−1.095311	−2.648278
29	1	0	−5.270484	0.613619	−2.670087
30	6	0	−2.554512	0.943856	1.619152
31	1	0	−3.567044	0.980999	2.023348
32	1	0	−2.320832	1.917769	1.187012
33	1	0	−1.886739	0.791691	2.474808
34	6	0	1.103700	0.531729	−1.150524
35	1	0	1.416940	1.049072	−2.057751
36	6	0	2.008344	−0.140498	−0.419844
37	6	0	1.567763	−0.807737	0.819434
38	6	0	0.154893	−0.545515	1.354940
39	1	0	0.237915	0.406422	1.909294
40	8	0	−0.173308	−1.582888	2.250753
41	1	0	0.689145	−1.950796	2.529973
42	8	0	2.311532	−1.549038	1.456050
43	1	0	−2.905953	−0.935923	−1.322895
44	8	0	−0.302132	2.081475	−0.077304
45	1	0	0.352615	2.071865	0.645786
46	6	0	3.490035	−0.142032	−0.727681
47	1	0	3.624696	0.217006	−1.757225
48	6	0	4.257620	0.818981	0.201541
49	1	0	5.328178	0.739021	0.017645
50	1	0	4.047481	0.610546	1.250896
51	6	0	4.143086	−1.525045	−0.670363
52	8	0	5.245602	−1.745364	−0.220140
53	8	0	3.367387	−2.451416	−1.261930
54	6	0	3.888856	−3.792063	−1.239516
55	1	0	4.009299	−4.130617	−0.207481
56	1	0	4.854768	−3.837418	−1.748966
57	1	0	3.149161	−4.400624	−1.760071

58	8	0	3.913420	2.190394	−0.110116
59	6	0	2.913641	2.776067	0.574514
60	6	0	2.631112	4.169081	0.071372
61	1	0	1.683480	4.136264	−0.479230
62	1	0	3.421854	4.538599	−0.582893
63	1	0	2.494099	4.839493	0.923784
64	8	0	2.282875	2.234020	1.466285
Conformer	**1d−2**				
Center	Atomic	Atomic	Coordinates (Angstroms)		
Number	Number	Type	X	Y	Z
1	6	0	−4.929492	1.835740	−0.429367
2	1	0	−5.215924	2.892735	−0.351245
3	1	0	−5.362118	1.481399	−1.373955
4	6	0	−5.517401	1.060087	0.750219
5	1	0	−6.614379	1.127780	0.750043
6	1	0	−5.180955	1.541476	1.681077
7	6	0	−5.101161	−0.431277	0.798575
8	6	0	−3.539956	−0.524567	0.641747
9	6	0	−2.882004	0.270243	−0.554706
10	6	0	−3.399484	1.735206	−0.467830
11	1	0	−2.997695	2.199034	0.445918
12	1	0	−3.003537	2.315681	−1.301526
13	6	0	−2.990663	−1.958296	0.743800
14	1	0	−3.492162	−2.508499	1.545746
15	1	0	−3.172545	−2.515730	−0.181398
16	6	0	−1.490166	−1.928735	1.034169
17	1	0	−1.079553	−2.947612	1.106969
18	1	0	−1.308002	−1.452050	2.007638
19	6	0	−0.717761	−1.165595	−0.047331
20	6	0	−1.322865	0.250465	−0.295993
21	1	0	−1.168255	0.800413	0.643271
22	6	0	−5.897811	−1.251689	−0.239261
23	1	0	−5.832516	−0.847502	−1.251276
24	1	0	−6.959461	−1.263726	0.037772
25	1	0	−5.559359	−2.293461	−0.275229
26	6	0	−5.502592	−0.960505	2.196761
27	1	0	−6.553831	−0.719736	2.396917
28	1	0	−4.900408	−0.499128	2.989393
29	1	0	−5.400631	−2.047740	2.283102
30	6	0	−3.198903	−0.284538	−1.962440
31	1	0	−4.246697	−0.136113	−2.227597
32	1	0	−2.964985	−1.345142	−2.064014

33	1	0	−2.609016	0.254872	−2.711125
34	6	0	0.750389	−1.068684	0.330636
35	1	0	1.151312	−1.896871	0.917085
36	6	0	1.569971	−0.072478	−0.043373
37	6	0	1.013793	1.037047	−0.840539
38	6	0	−0.418508	0.920888	−1.365517
39	1	0	−0.364597	0.238822	−2.229960
40	8	0	−0.846264	2.196794	−1.781329
41	1	0	−0.028019	2.729794	−1.841820
42	8	0	1.672632	2.038802	−1.111497
43	1	0	−3.166083	0.000753	1.537723
44	8	0	−0.786844	−1.900547	−1.294138
45	1	0	−0.378766	−2.769095	−1.142541
46	6	0	3.043125	−0.024779	0.320448
47	1	0	3.235411	−0.876629	0.981876
48	6	0	3.934168	−0.199268	−0.923658
49	1	0	4.026405	0.733772	−1.476273
50	1	0	3.509944	−0.977994	−1.561903
51	6	0	3.323483	1.228552	1.157913
52	8	0	2.628908	1.565897	2.092050
53	8	0	4.433772	1.880521	0.773305
54	6	0	4.732726	3.073541	1.517575
55	1	0	5.658840	3.458485	1.090186
56	1	0	4.862108	2.842161	2.577975
57	1	0	3.924348	3.800474	1.405824
58	8	0	5.284103	−0.571022	−0.577399
59	6	0	5.486994	−1.873761	−0.270963
60	6	0	6.945377	−2.133262	0.022377
61	1	0	7.082311	−3.181967	0.287825
62	1	0	7.284112	−1.491662	0.842266
63	1	0	7.553588	−1.888362	−0.854614
64	8	0	4.604137	−2.706024	−0.241439
Conformer	**1d−3**				
Center	Atomic	Atomic	Coordinates (Angstroms)		
Number	Number	Type	X	Y	Z
1	6	0	−4.879125	1.805304	−0.966670
2	1	0	−5.104288	2.869039	−1.118163
3	1	0	−5.280178	1.289211	−1.848268
4	6	0	−5.576197	1.311817	0.302126
5	1	0	−6.665257	1.433015	0.217880
6	1	0	−5.260623	1.951892	1.140080
7	6	0	−5.256111	−0.158407	0.669537

8	6	0	−3.697169	−0.355255	0.639613
9	6	0	−2.927767	0.147562	−0.645693
10	6	0	−3.358343	1.623501	−0.884528
11	1	0	−2.979211	2.240457	−0.055568
12	1	0	−2.882255	2.004336	−1.788357
13	6	0	−3.240091	−1.760740	1.067954
14	1	0	−3.816987	−2.108828	1.930181
15	1	0	−3.402073	−2.489106	0.266000
16	6	0	−1.759290	−1.738810	1.447125
17	1	0	−1.412957	−2.738007	1.754864
18	1	0	−1.605948	−1.079872	2.313273
19	6	0	−0.883559	−1.251565	0.287837
20	6	0	−1.389280	0.108433	−0.285029
21	1	0	−1.254538	0.833625	0.530314
22	6	0	−6.040933	−1.129520	−0.239097
23	1	0	−5.893614	−0.939579	−1.303997
24	1	0	−7.115355	−1.034421	−0.038038
25	1	0	−5.765616	−2.172770	−0.046685
26	6	0	−5.768218	−0.378010	2.113739
27	1	0	−6.809389	−0.042296	2.194103
28	1	0	−5.178991	0.194410	2.841053
29	1	0	−5.747411	−1.431108	2.413845
30	6	0	−3.198512	−0.664438	−1.932884
31	1	0	−3.036353	−1.735008	−1.801399
32	1	0	−2.527670	−0.326110	−2.729646
33	1	0	−4.215060	−0.512711	−2.299230
34	6	0	0.564676	−1.144330	0.736684
35	1	0	0.878322	−1.844417	1.513156
36	6	0	1.462194	−0.287889	0.222164
37	6	0	1.012860	0.662915	−0.814568
38	6	0	−0.387450	0.497697	−1.407079
39	1	0	−0.322384	−0.352995	−2.104737
40	8	0	−0.717632	1.673369	−2.107775
41	1	0	0.128087	2.155212	−2.207653
42	8	0	1.732832	1.572526	−1.218345
43	1	0	−3.343444	0.324761	1.434064
44	8	0	−0.925903	−2.223602	−0.785453
45	1	0	−0.626499	−3.072790	−0.421049
46	6	0	2.909908	−0.219769	0.676606
47	1	0	3.025301	−0.926643	1.508077
48	6	0	3.882353	−0.650717	−0.427014
49	1	0	4.051822	0.141389	−1.156154

50	1	0	3.496343	−1.530865	−0.952022
51	6	0	3.185906	1.164899	1.281921
52	8	0	2.460204	1.675651	2.107217
53	8	0	4.318248	1.723771	0.826040
54	6	0	4.603732	3.036231	1.339348
55	1	0	5.544489	3.331785	0.874547
56	1	0	4.700442	3.009224	2.427728
57	1	0	3.804300	3.729680	1.066947
58	8	0	5.123027	−1.001937	0.217571
59	6	0	6.159889	−1.275235	−0.610394
60	6	0	7.409596	−1.581824	0.181640
61	1	0	7.225169	−2.404122	0.880457
62	1	0	7.698951	−0.707782	0.774670
63	1	0	8.216224	−1.847525	−0.502315
64	8	0	6.072005	−1.267551	−1.817753

Table S9. DP4+ analysis of compound **1**

		DP4+	0. 01%	99. 99%	0. 00%	0. 00%
Nuclei	sp2?	xperimenta	Isomer 1	Isomer 2	Isomer 3	Isomer 4
C		39. 7	39. 95	41. 58	42. 35	42. 24
C		18. 3	21. 22	21. 18	21. 06	20. 78
C		41. 2	42. 46	42. 18	43. 24	42. 58
C		33. 1	38. 11	38. 24	38. 33	38. 22
C		54. 2	55. 85	56. 87	58. 54	58. 32
C		19	21. 04	21. 69	20. 44	20. 25
C		41. 4	42. 88	43. 72	43. 86	43. 13
C		69. 4	73. 09	72. 19	74. 48	74. 95
C		60. 2	62. 19	62. 58	63. 1	61. 42
C		37. 5	42. 15	42. 34	45. 66	45. 18
C		71. 8	75. 96	77. 01	77. 6	76. 23
C	x	195. 8	205. 7	206. 35	212. 07	211. 77
C	x	133. 6	140. 22	140. 44	138. 17	138. 12
C	x	154	170. 31	166. 79	165. 03	163. 53
C		44. 9	48. 54	49. 32	47. 19	52. 5
C		62. 4	65. 1	67. 53	67. 79	65. 72
C	x	170. 9	179. 79	180. 24	179. 51	178. 79
C		33. 8	33. 57	33. 48	33. 42	33. 54
C		22	21. 58	21. 86	22. 04	21. 89
C		17. 7	19. 72	18. 87	15. 67	15. 68
C		52. 3	53. 61	54. 16	54. 31	53. 79
C	x	170. 7	180. 41	181. 01	181. 24	183. 87
C		20. 8	23. 12	23. 05	22. 69	23. 43

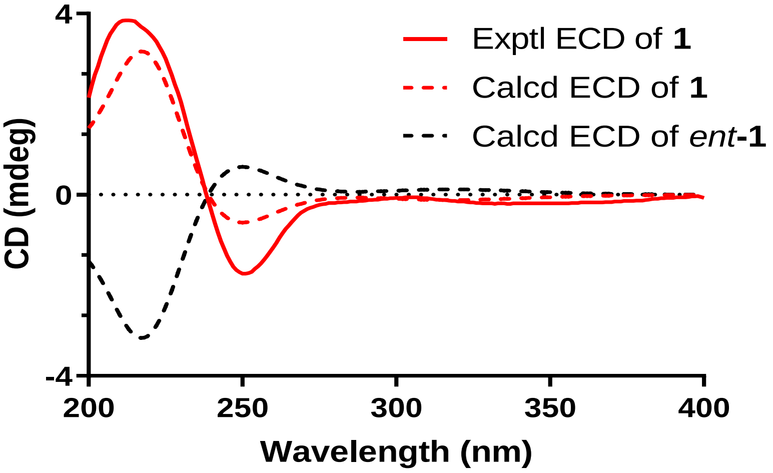

Figure S18. Experimental and calcultaed ECD spectra of **1**

NMR and ECD calculation method of compound 2

Calculation details

The random conformational searches were performed by SYBYL X 2.1.1 program using MMFF94s molecular force field. The obtained conformers were subsequently optimized by using Gaussion09 software at the B3LYP/6−31G(d) level in gas phase. The optimized stable conformers were selected for further NMR calculations at the mPW1PW91/6−31+G(d,p) level in chloroform and ECD calculations at the cam−B3LYP/6−31+G(d) level in acetonitrile. The overall theoretical NMR data were analyzed by using linear regression. The overall ECD data were weighted by Boltzmann distribution and produced by SpecDis 1.70.1 software.

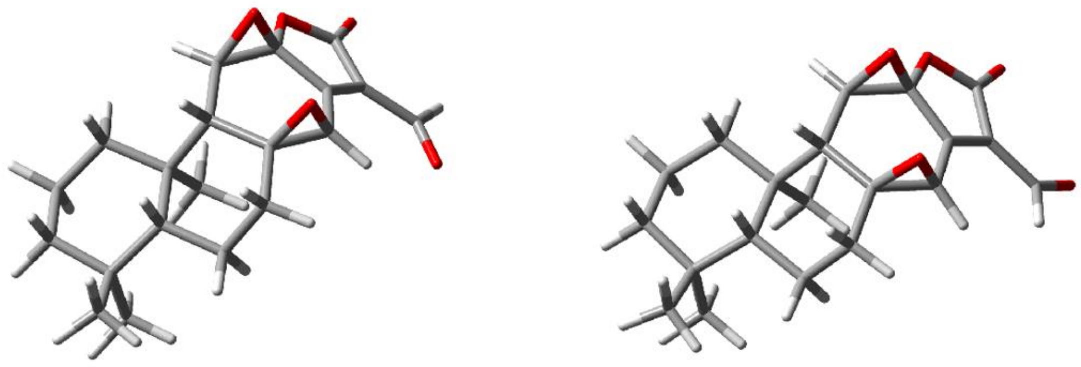

98.78 %　　　　1.22 %

Figure S19. Optimized geometries of dominant conformers of conformer **2a** and **2b**

Table S10. Energy analysis of **2**

conformers	B3LYP/6−31G(d) Gibbs free energy (298.15 K)		
	Energy (Hartree)	Energy (kcal/mol)	Population (%)
**2a**	−1152.449824	−723173.1769	98.78
**2b**	−1152.445677	−723170.5747	1.22

Table S11. Cartesian coordinates of optimized **2**

Conformer **2a**					
Center	Atomic	Atomic	Coordinates (Angstroms)		
Number	Number	Type	X	Y	Z
1	6	0	−2.494048	0.477197	−0.216012
2	6	0	−1.355687	−0.604872	−0.085960
3	6	0	−1.930847	1.894669	−0.006165
4	1	0	−2.728512	2.639791	−0.080969

5	1	0	−1.506187	2.001366	0.998569
6	6	0	−0.869064	2.216610	−1.061601
7	1	0	−0.406610	3.193775	−0.884462
8	1	0	−1.347331	2.273844	−2.050172
9	6	0	0.215861	1.161255	−1.160429
10	6	0	−0.286158	−0.296396	−1.212764
11	6	0	1.599761	1.498960	−0.674870
12	1	0	1.792634	2.468156	−0.224148
13	6	0	2.462974	0.392707	−0.264080
14	6	0	2.201865	−0.965811	−0.791702
15	6	0	0.837349	−1.331349	−1.207627
16	1	0	0.552441	−2.371507	−1.073115
17	1	0	−2.789833	0.423995	−1.277623
18	6	0	−0.641702	−0.611156	1.286573
19	1	0	−1.323185	−0.851188	2.103355
20	1	0	−0.175912	0.349761	1.524127
21	1	0	0.149305	−1.369472	1.303618
22	1	0	−0.799998	−0.396076	−2.181577
23	6	0	−1.975705	−2.003484	−0.351205
24	1	0	−2.254793	−2.072225	−1.413463
25	1	0	−1.235074	−2.791267	−0.171992
26	6	0	−3.219201	−2.293790	0.497835
27	1	0	−3.606496	−3.286953	0.238329
28	1	0	−2.951367	−2.345272	1.560841
29	6	0	−4.302324	−1.239865	0.266025
30	1	0	−5.189345	−1.460969	0.874729
31	1	0	−4.627279	−1.301455	−0.783527
32	6	0	−3.839108	0.209535	0.553342
33	6	0	−3.769437	0.454122	2.076914
34	1	0	−3.363639	1.443695	2.313265
35	1	0	−3.167969	−0.287265	2.608036
36	1	0	−4.779814	0.409530	2.501241
37	6	0	−4.930736	1.151736	−0.007791
38	1	0	−4.773500	2.196430	0.280424
39	1	0	−5.913125	0.855865	0.379259
40	1	0	−4.978681	1.105291	−1.102711
41	8	0	1.249632	1.515751	−2.074158
42	6	0	3.434337	0.290031	0.674319
43	6	0	3.862967	−1.131569	0.739337
44	8	0	3.079565	−1.854001	−0.156643
45	8	0	4.700742	−1.650244	1.427828
46	8	0	1.929938	−1.181535	−2.148289

47	6	0	4.010087	1.337159	1.532326
48	1	0	4.853977	1.006291	2.166559
49	8	0	3.603022	2.486496	1.560339
Conformer **2b**					
Center	Atomic	Atomic	Coordinates (Angstroms)		
Number	Number	Type	X	Y	Z
1	6	0	−2.564536	0.501158	−0.136563
2	6	0	−1.410419	−0.571139	−0.112714
3	6	0	−2.011449	1.906933	0.161238
4	1	0	−2.818201	2.646045	0.159502
5	1	0	−1.563467	1.946350	1.161004
6	6	0	−0.979448	2.315865	−0.893582
7	1	0	−0.526746	3.286435	−0.660898
8	1	0	−1.482067	2.434229	−1.864202
9	6	0	0.116106	1.285673	−1.091737
10	6	0	−0.369275	−0.170215	−1.237641
11	6	0	1.506737	1.605383	−0.623459
12	1	0	1.676181	2.552731	−0.114042
13	6	0	2.412222	0.493421	−0.315446
14	6	0	2.136056	−0.831978	−0.917604
15	6	0	0.767817	−1.184965	−1.327464
16	1	0	0.498047	−2.235595	−1.260528
17	1	0	−2.887493	0.520870	−1.191445
18	6	0	−0.664611	−0.666614	1.238947
19	1	0	−0.200195	0.279898	1.531412
20	1	0	0.132101	−1.416944	1.185740
21	1	0	−1.325365	−0.965994	2.052675
22	1	0	−0.902716	−0.207765	−2.200161
23	6	0	−2.020128	−1.955296	−0.465995
24	1	0	−2.324781	−1.948330	−1.523431
25	1	0	−1.265702	−2.743820	−0.364897
26	6	0	−3.237735	−2.324515	0.388907
27	1	0	−3.619658	−3.301053	0.066264
28	1	0	−2.942280	−2.449969	1.438208
29	6	0	−4.338786	−1.271218	0.262028
30	1	0	−5.207741	−1.548922	0.873465
31	1	0	−4.688765	−1.259778	−0.781203
32	6	0	−3.886202	0.159250	0.644437
33	6	0	−3.781300	0.292333	2.180076
34	1	0	−3.163020	−0.481113	2.642116
35	1	0	−4.781013	0.208559	2.623223
36	1	0	−3.376314	1.265454	2.478311

37	6	0	−5.002828	1.124624	0.180194
38	1	0	−5.971248	0.788815	0.569837
39	1	0	−5.078755	1.155711	−0.913703
40	1	0	−4.850687	2.148147	0.538450
41	8	0	1.123321	1.725205	−2.004917
42	6	0	3.432935	0.350401	0.562978
43	6	0	3.868544	−1.080633	0.534472
44	8	0	3.036137	−1.747291	−0.373138
45	8	0	4.725765	−1.654001	1.140852
46	8	0	1.831549	−0.951207	−2.282802
47	6	0	4.024016	1.393163	1.419816
48	1	0	3.578375	2.402795	1.283072
49	8	0	4.921380	1.213976	2.215743

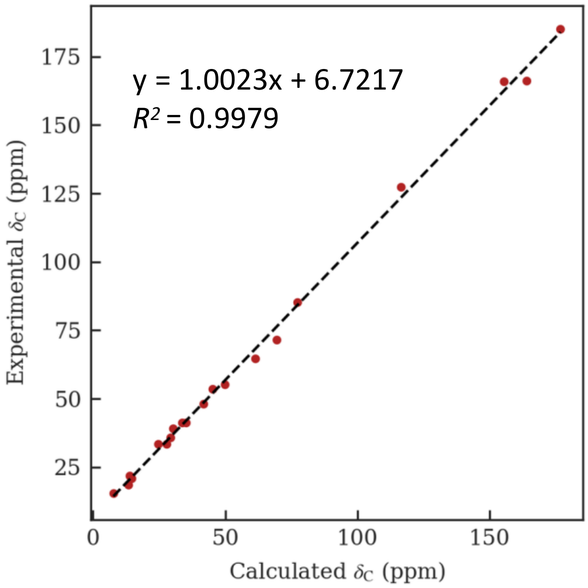

Figure S20. Linear correlation plots of predicted versus experimental ^{13}C NMR chemical shifts

Table S12. Parameters of the calculated ^{13}C NMR chemical shifts of **2**

CMAD	CLAD	R^2	RMSD	F	p
1.88	4.79	0.9979	2.4544	8723.15	< 0.01

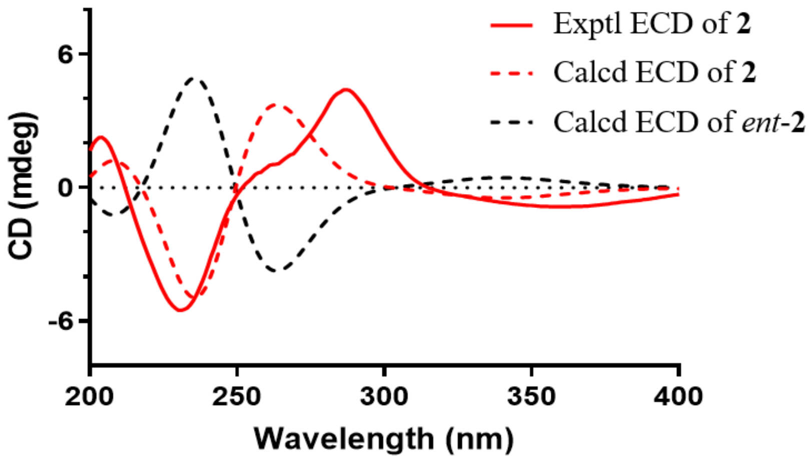

Figure S21. Experimental and calculated ECD spectra of **2**

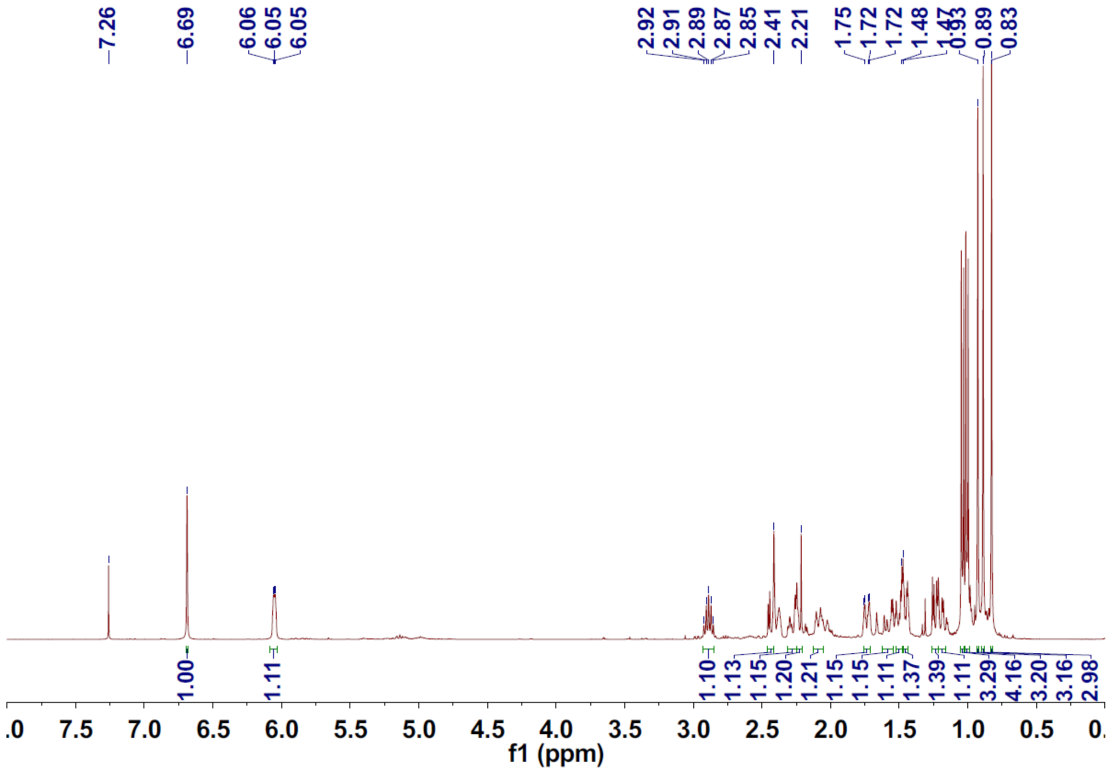

Figure S22. ^{1}H NMR spectrum of **3** in $CDCl_3$

Elmt	Val.	Min	Max	Elmt	Val.	Min	Max	Elmt	Val.	Min	Max	Elmt	Val.	Min	Max	Use Adduct
H	1	0	40	O	2	1	20	K	1	0	0	Pd	2	0	0	H
2H	1	0	0	F	1	0	0	Mn	3	0	0	I	3	0	0	Na
3H	1	0	0	Si	4	0	0	Fe	2	0	0	Pt	2	0	0	
B	3	0	0	P	3	0	0	Cu	2	0	0	Au	1	0	0	
C	4	10	30	S	2	0	0	Se	2	0	0					
N	3	0	0	Cl	1	0	0	Br	1	0	0					

Error Margin (ppm): 500
HC Ratio: 0.0 - 20.0
Max Isotopes: all
MSn Iso RI (%): 75.00

DBE Range: -100.0 - 1000.0
Apply N Rule: yes
Isotope RI (%): 1.00
MSn Logic Mode: AND

Electron Ions: both
Use MSn Info: no
Isotope Res: 10000
Max Results: 2000

Event#: 1 MS(E+) Ret. Time : 2.253 Scan# : 339

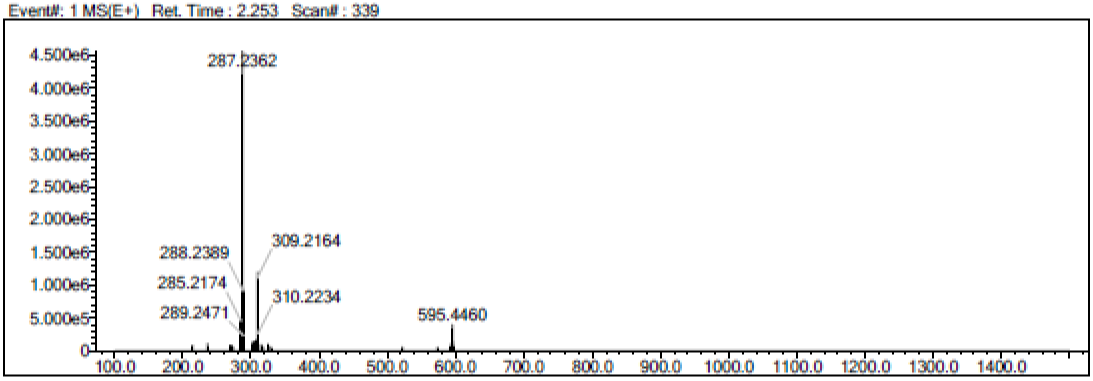

Measured region for 287.2362 m/z

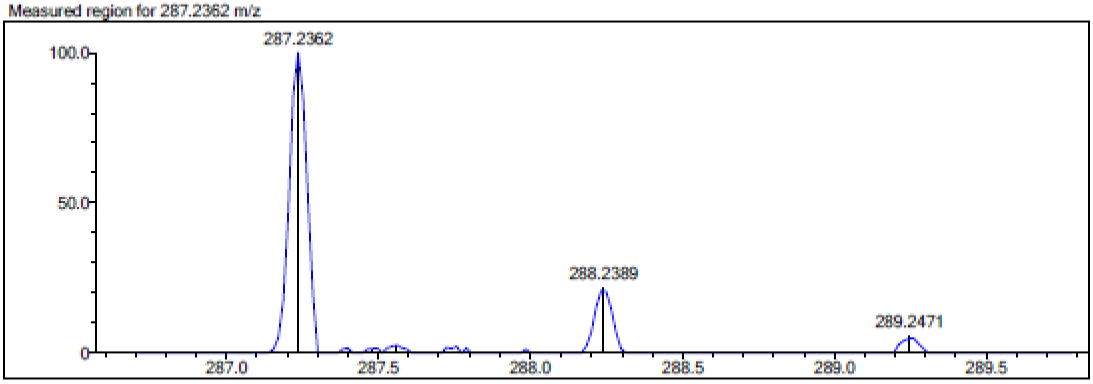

C20 H30 O [M+H]+ : Predicted region for 287.2369 m/z

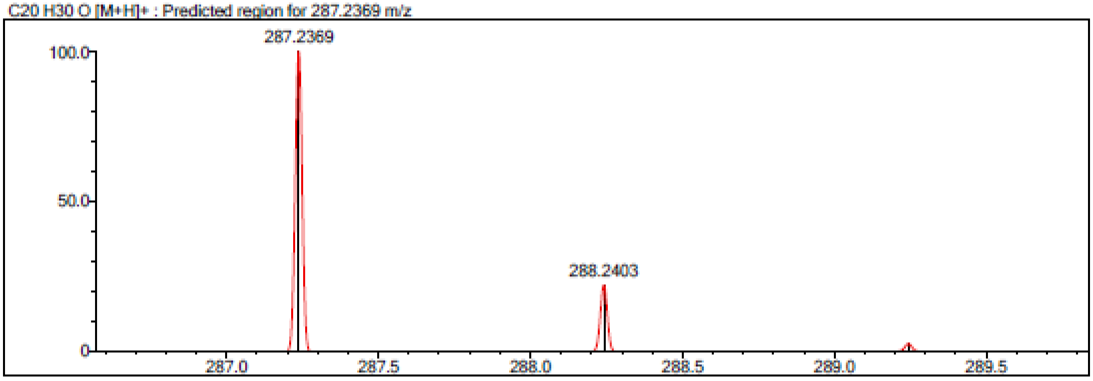

Rank	Score	Formula (M)	Ion	Meas. m/z	Pred. m/z	Df. (mDa)	Df. (ppm)	Iso	DBE
1	76.95	C20 H30 O	[M+H]+	287.2362	287.2369	-0.7	-2.44	79.83	6.0

Figure S23. HRESIMS spectrum of **3**

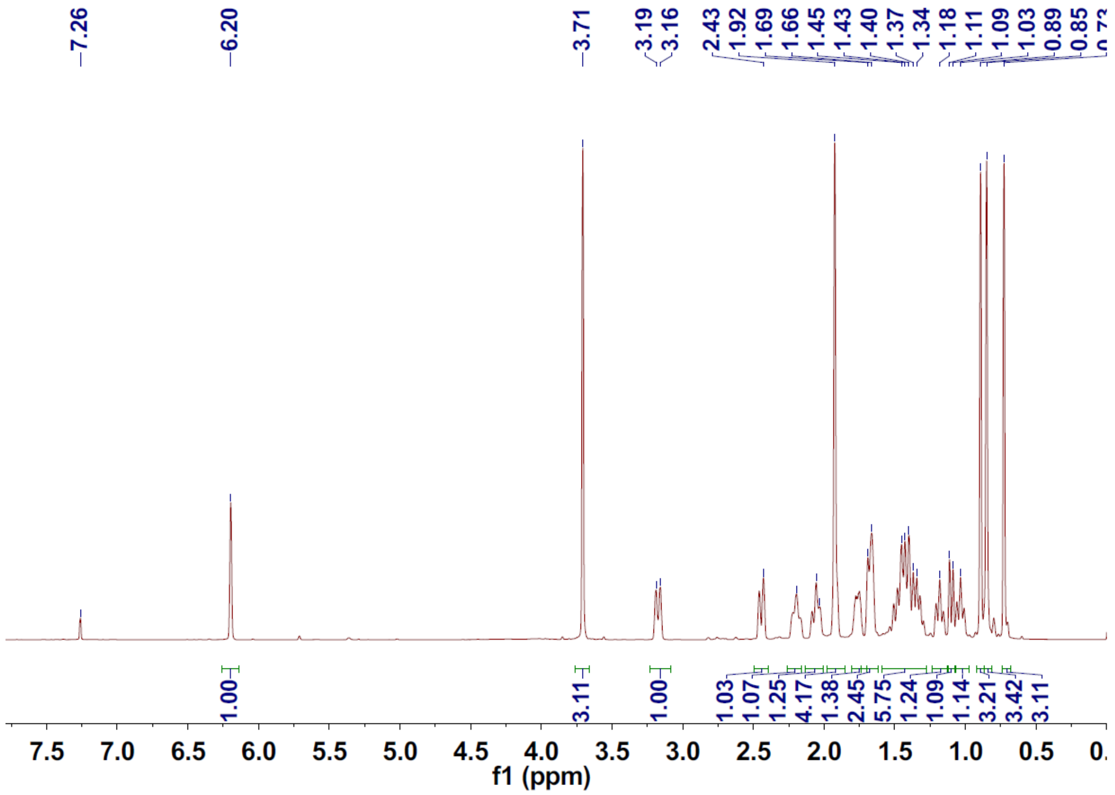

Figure S24. ^{1}H NMR spectrum of **4** in $CDCl_3$

Elmt	Val.	Min	Max	Elmt	Val.	Min	Max	Elmt	Val.	Min	Max	Elmt	Val.	Min	Max	Use Adduct
H	1	1	40	O	2	0	10	K	1	0	0	Pd	2	0	0	H
2H	1	0	0	F	1	0	0	Mn	3	0	0	I	3	0	0	Na
3H	1	0	0	Si	4	0	0	Fe	2	0	0	Pt	2	0	0	K
B	3	0	0	P	3	0	0	Cu	2	0	0	Au	1	0	0	NH4
C	4	0	30	S	2	0	0	Se	2	0	0					
N	3	0	0	Cl	1	0	0	Br	1	0	0					

Error Margin (ppm):	100	DBE Range:	-100.0 - 100.0	Electron Ions:	both
HC Ratio:	0.0 - 20.0	Apply N Rule:	yes	Use MSn Info:	no
Max Isotopes:	all	Isotope RI (%):	1.00	Isotope Res:	10000
MSn Iso RI (%):	75.00	MSn Logic Mode:	AND	Max Results:	2000

Event#: 1 MS(E+) Ret. Time : 3.333 Scan# : 501

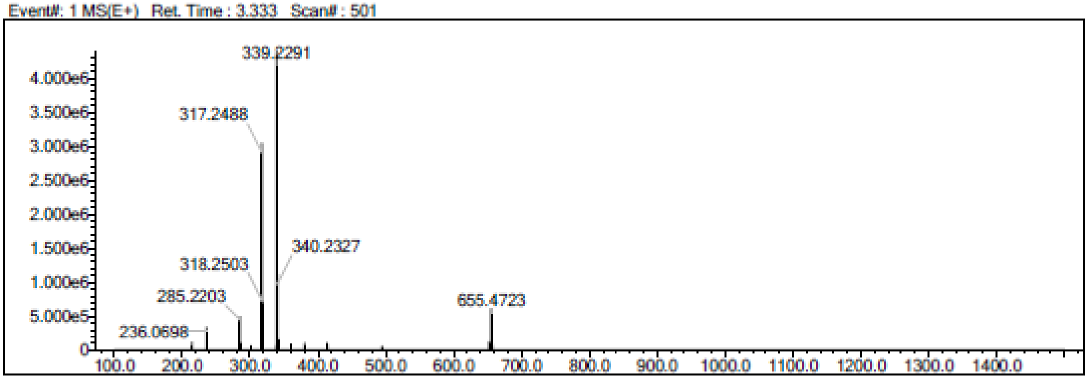

Measured region for 339.2291 m/z

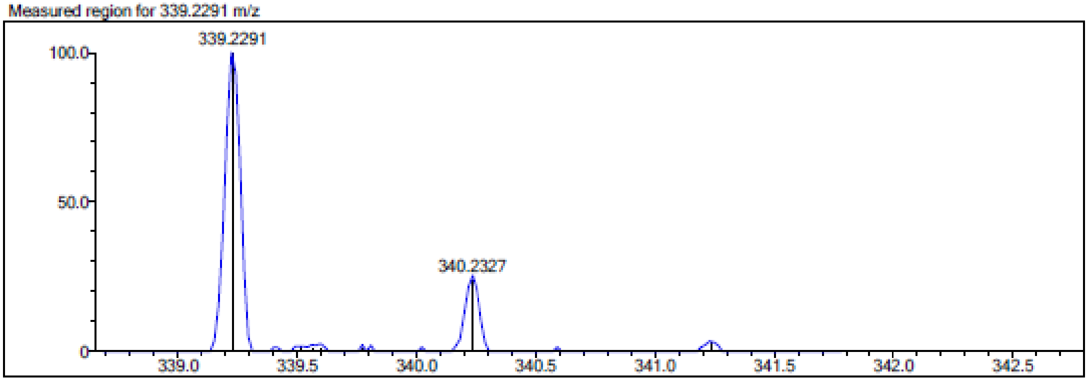

C21 H32 O2 [M+Na]+ : Predicted region for 339.2295 m/z

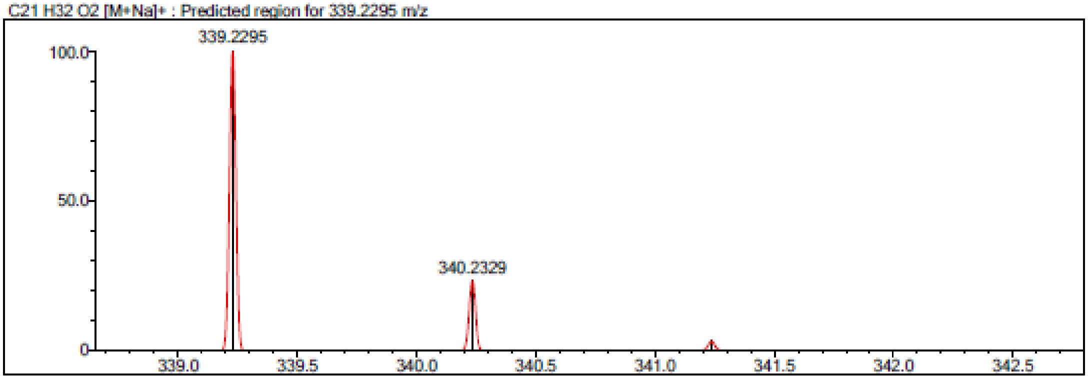

Rank	Score	Formula (M)	Ion	Meas. m/z	Pred. m/z	Df. (mDa)	Df. (ppm)	Iso	DBE
1	79.26	C21 H32 O2	[M+Na]+	339.2291	339.2295	-0.4	-1.18	79.62	6.0

Figure S25. HRESIMS spectrum of **4**

Table S13. NMR Data for compounds **1− 2** in $CDCl_3$

Position	1			2		
	δ_H (*J* in Hz)	δ_C, type	HMBC	δ_H (*J* in Hz)	δ_C, type	HMBC
1	1.88 (1H, d, *J* = 13.0 Hz)<br>1.21−1.16 (1H, m)	39.7, CH_2	3, 5, 20	1.92 (1H, d, *J* = 12.5 Hz)<br>1.37−1.29 (1H, m)	39.2, CH_2	3, 5, 20
2	1.55 (1H, d, *J* = 13.5 Hz)<br>1.51−1.45 (1H, m)	18.5, CH_2	1, 3, 4	1.63−1.55 (1H, m)<br>1.56−1.51 (1H, m)	18.5, CH_2	1, 3, 4
3	1.43 (1H, d, *J* = 12.7 Hz)<br>1.26−1.19 (1H, m)	41.6, CH_2	1, 2, 4, 5, 18, 19	1.47−1.43 (1H, m)<br>1.29−1.20 (1H, m)	41.4, CH_2	1, 2, 4, 5, 19, 18,
4		33.3, C			33.6, C	
5	1.08 (1H, dd, *J* = 12.9, 2.6 Hz)	54.4, CH	1, 3, 4, 6, 7, 9, 10, 18, 19,20	1.12 (1H, dd, *J* = 12.3, 2.5 Hz)	53.6, CH	3, 4, 6,7, 9, 10, 20
6	1.71−1.65 (1H, m)<br>1.16−1.12 (1H, m)	19.2, CH_2	4, 5, 7, 8, 10	1.88−1.80 (1H, m)<br>1.56−1.51 (1H, m), overlapped	20.9, CH_2	4, 5, 8, 10
7	2.13−2.03(1H, m)<br>1.74−1.72 (1H, m)	41.4, CH_2	5, 6, 8, 9, 14	2.08−1.97 (1H, m)<br>1.51−1.48 (1H, m), overlapped	35.8, CH_2	5, 6, 8, 9, 10, 14,
8		69.5, C			71.7, C	
9	2.08 (1H, s)	60.4, CH	1, 8, 10, 11, 12, 14, 20	2.33 (1H, s)	48.1, CH	5, 12, 13, 14
10		37.7, C			39.4, C	
11	4.23 (1H, s)	72.0, CH	8, 9, 10, 12, 13	4.15 (1H, s)	64.8, CH	8, 9, 10, 12
12		196.0, C			85.4, C	
13		133.7, C			166.2, C	
14	6.71 (1H, s)	154.2, CH	7, 8. 9, 12, 13 15	4.48 (1H, s)	55.3, CH	7, 8, 12, 13
15	3.77 (1H, t, *J* = 7.2 Hz)	45.7, CH	12, 13, 14, 16, 17		127.5, C	
16		170.8, C			165.8, C	
17	4.41 (2H, d, *J* = 7.2 Hz)	62.5, CH_2	13,15, 16, 1′	9.97 (1H, s)	185.0, CH	13, 15
18	0.91 (3H, s)	34.0, CH_3	3, 4, 5	0.94 (3H, s)	33.6, CH_3	3, 4, 5, 19
19	0.79 (3H, s)	22.1, CH_3	3, 4, 5, 18	0.85 (3H, s)	22.0, CH_3	3, 5, 18
20	0.69 (3H, s)	17.8, CH_3	1,5, 9, 10	0.80 (3H, s)	15.6, CH_3	1, 9
16−OCH_3	3.68 (3H, s)	52.4, CH_3	16			
1′		171.1, C				
2′	2.02 (3H, s)	21.0, CH_3	1′			
